# Activated STING in the thymic epithelium alters T cell development and selection leading to autoimmunity

**DOI:** 10.1172/JCI180252

**Published:** 2025-06-26

**Authors:** Zimu Deng, Christopher S. Law, Santosh Kurra, Noa Simchoni, Anthony K. Shum

**Affiliations:** 1 Department of Medicine and; 2 Cardiovascular Research Institute, UCSF, San Francisco, California, USA.

**Keywords:** Autoimmunity, Immunology, Autoimmune diseases, Innate immunity, Tolerance

## Abstract

Coatomer protein complex subunit α (COPA) syndrome is a monogenic disorder of immune dysregulation that leads to interstitial lung disease and high-titer autoantibodies. Constitutive activation of the innate immune molecule stimulator of interferon genes (STING) is centrally involved in disease. However, the mechanisms by which STING results in autoimmunity are not well understood in COPA syndrome and other STING-associated diseases. Prior studies showed a cell autonomous role for STING in thymocyte development. Single-cell data of human thymus demonstrated that STING is highly expressed in medullary thymic epithelial cells (mTECs) and at levels much greater than in T cells. Here, we show that in certain contexts, activated STING exerts a functional role in the thymic epithelium to alter thymocyte selection and predisposes to autoimmunity. In *Copa^E241K/+^* mice, activated STING in mTECs amplified IFN signaling, impaired macroautophagy, and caused a defect in negative selection of T cell precursors. WT mice given a systemic STING agonist phenocopied the selection defect and showed enhanced thymic escape of a T cell clone targeting a self-antigen also expressed in melanoma. Our work demonstrates that STING activation in TECs shapes the T cell repertoire and contributes to autoimmunity, findings that are important for conditions that activate thymic STING.

## Abstract 2

<span>Activation of STING in thymic epithelial cells shifts the T cell repertoire to promote autoimmunity demonstrating how loss of immune tolerance occurs in COPA syndrome and related disorders.</span>

## Graphical abstract

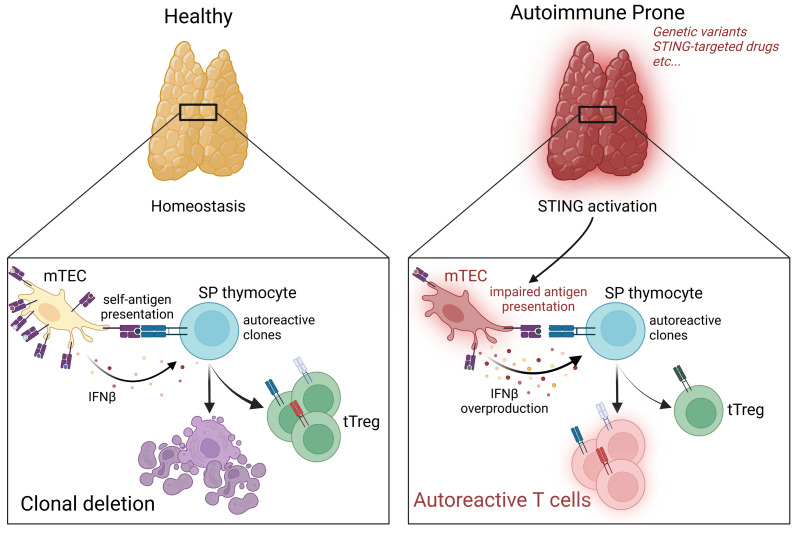

## Introduction

Coatomer protein complex subunit α (COPA) is a subunit of coat protein complex I that mediates protein transport from Golgi to ER. Monogenic missense mutations in the *COPA* gene have been implicated in COPA syndrome, a disorder of immune dysregulation that presents with interstitial lung disease, renal disease, and inflammation of small and large joints (1). We and others recently found that mutant COPA causes mis-trafficking of stimulator of interferon genes (STING), the key adaptor molecule of the cytosolic DNA sensing pathway in eukaryotic cells (2–4). In COPA syndrome, STING accumulates on the Golgi, where it persistently signals and leads to chronic inflammation (2).

COPA syndrome was originally described as an autoimmune disorder given the presence of very-high-titer autoantibodies, autoantibody-mediated organ infiltration, and increased levels of activated, cytokine-secreting CD4^+^ T cells in patients (1). Consistent with these findings, we observed that conventional T cells play a major role in driving organ autoimmunity in *Copa^E241K/+^* mice (5). Other studies of STING-associated interferonopathies, such as STING-associated vasculopathy with onset in infancy (SAVI) and Aicardi-Goutières syndrome, also show that T cells have a key pathogenic function in disease (6, 7). STING is known to have cell autonomous, multifaceted roles in regulating T cell differentiation and function. Agonists targeting the cGAS/STING pathway promote the stem cell–like differentiation of CD8^+^ T cells and enhance their cytolytic activity (8–10). Intrinsic STING activation in CD4^+^ T cells supports their differentiation to Th1 cells by IFN regulatory factors and IFN and to Th9 cells by mTOR (11). Multiple labs have shown that STING activation causes T cell death (7, 12–15), which may involve elevated ER stress acting downstream of T cell receptor (TCR) signaling (13). Taken together, these data underscore the unique nature by which STING impacts diverse intracellular pathways and mediates distinct responses in T cells.

Although many studies have demonstrated that STING regulates T cell function and homeostasis through cell-intrinsic effects, whether activation of STING in thymic epithelial cells also influences T cell development remains unexplored. In previous work, we found that *Copa^E241K/+^* mice have a defect in thymocyte negative selection, which maps to the thymic stroma, as well as elevated levels of STING-dependent type 1 IFNs in medullary thymic epithelial cells (mTECs). In this study, we suggest that STING activation in the thymic epithelium contributes to a break in self-tolerance by directly altering T cell development and selection and that it is an important factor in triggering autoimmunity in COPA syndrome.

## Results

### Activated STING in thymic stroma of Copa^E241K/+^ mice upregulates IFNs.

**Figure 1:**
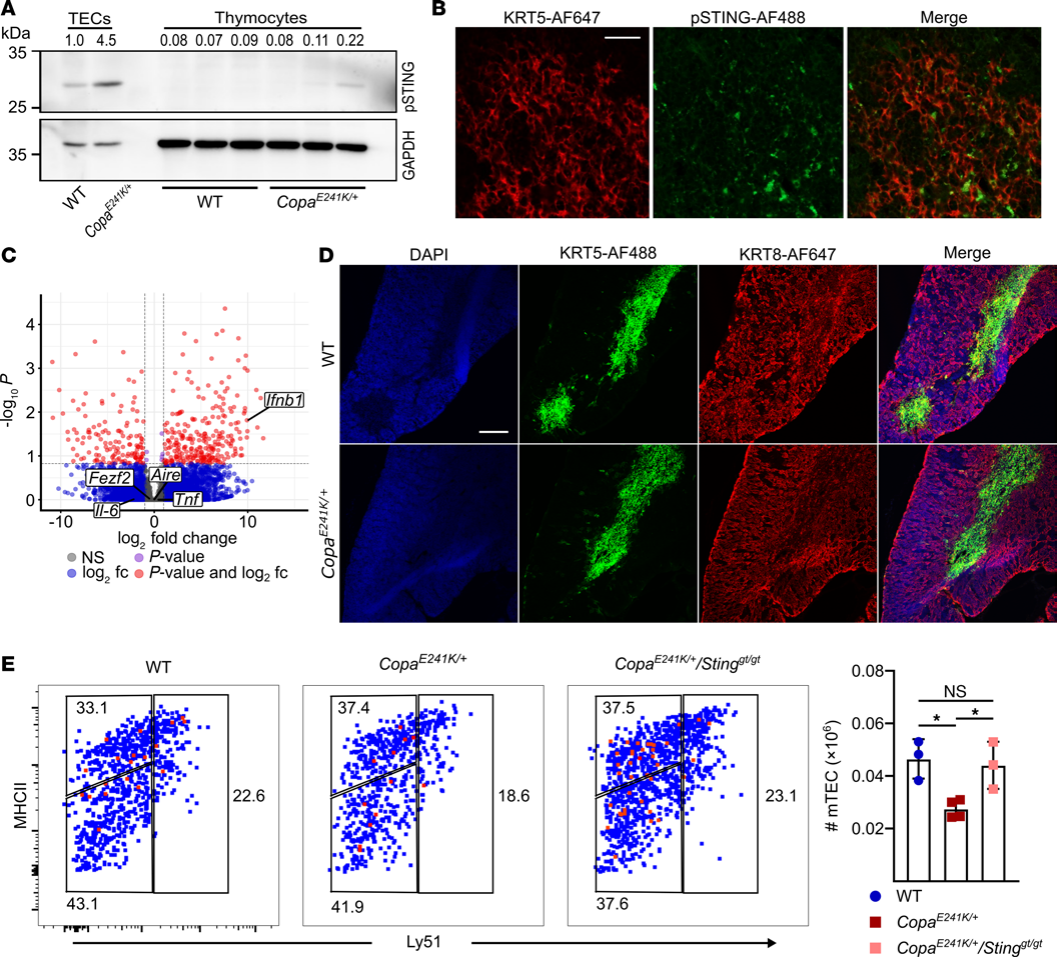
Activated STING in thymic stroma upregulates IFN signaling. (**A**) Representative immunoblot of pSTING in TECs versus thymocytes with relative band density quantification. Each lane represents 1 biological replicate. Data represent 3 independent experiments. (**B**) Immunofluorescence stain of keratin 5 (KRT5) and pSTING expression on thymic sections from *Copa^E241K/+^* mice. Scale bar: 100 μm. (**C**) Volcano plot of RNA sequencing analysis of sorted mTECs (mTEC^hi^) from WT and *Copa^E241K/+^* mice (*n* = 3 per genotype). (**D**) Immunofluorescence stain of KRT5 and KRT8 on thymic sections from *Copa^E241K/+^* and WT littermates. Scale bar: 250 μm. Data represent 3 independent experiments. (**E**) Left: representative flow cytometry analysis of TECs via MHC-II and Ly51 staining. Right: total mTEC numbers (WT, *n* = 3; *Copa^E241K/+^*, *n* = 4; *Copa^E241K/+^*/*Sting^gt/gt^*, *n* = 3). Data represent 2 independent experiments. Data are mean ± SD. One-way ANOVA with Bonferroni’s multiple-comparison test was used for statistical analysis. A *P* value of less than 0.05 was considered statistically significant.

To assess whether STING has a functional role in the thymic stroma of *Copa^E241K/+^* mice that influences the development and selection of autoreactive T cells, we first determined whether we could detect activation of STING protein in TECs. We performed Western blots of enriched TEC cell populations and assayed for phosphorylated STING (pSTING). We found a significant increase in pSTING in TECs from *Copa^E241K/+^* mice in comparison with WT mice (Figure 1A and Supplemental Figure 1A; supplemental material available online with this article; https://doi.org/10.1172/JCI180252DS1) and used confocal microscopy to observe that pSTING was predominantly enriched in mTECs (Figure 1B and Supplemental Figure 1B). Consistent with these results, our previous bulk RNA sequencing data from sorted TEC populations of *Copa^E241K/+^* mice showed an increase in *Ifnb1* transcript levels, particularly in MHC-II^hi^CD80^hi^ mTECs (mTEC^hi^) (2). We performed deeper analysis of mTEC^hi^ cell bulk RNA sequencing data to determine whether other signaling outputs of STING were upregulated. Interestingly, although *Ifnb1* transcript was elevated in *Copa^E241K/+^* mice, the cytokines *Il6* and *Tnf* that are downstream of NF-κB signaling were not (Figure 1C). Similar results were obtained when we examined IL6 and TNF-α by flow cytometry in mTEC^hi^ cells from *Copa^E241K/+^* mice, both of which stayed largely normal, whereas the IFN-stimulated chemokine CCL5 was significantly elevated (Supplemental Figure 1, C and D).

Because activated STING is also associated with cellular senescence (16) and apoptosis (17), we examined tissue architecture of thymi by confocal microscopy to assess whether there was evidence of thymic involution. Immunofluorescence staining with KRT5 and KRT8 cell surface markers revealed that *Copa^E241K/+^* mice appeared to have normal size and organization of the cortical and medullary thymic epithelium (18) (Figure 1D). Measurement of a cellular senescence marker by flow cytometry appeared unchanged in mTECs between *Copa^E241K/+^* mice and controls (Supplemental Figure 3A). In contrast, we observed that *Copa^E241K/+^* mice had elevated annexin V staining in mTECs and a mild decrease in mTEC numbers that was reversed in the absence of STING (Supplemental Figure 3B and Figure 1E). Double positive (DP) thymocytes also had an increase in annexin V, although the cell counts remained largely normal (Supplemental Figure 3B and Supplemental Figure 4C). The mRNA levels of *Aire* and *Fezf2*, 2 key transcriptional regulators of tissue-specific antigen expression in the thymus, were similar in mTECs and other antigen presenting cells (APCs) in *Copa^E241K/+^* mice and controls (Figure 1C and Supplemental Figure 2E). Taken together, activated STING in the thymic stroma appeared functionally active, particularly within mTECs, and caused both an increase in mTEC IFN levels and a mild decrease in the cellularity of the mTEC compartment.

### Activated STING in thymic stroma increases postselection thymocytes.

**Figure 2:**
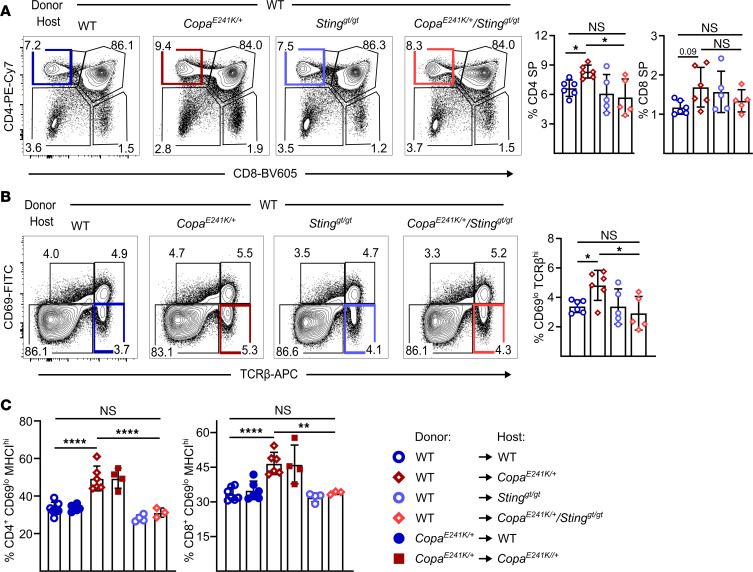
Activated STING in thymic stroma increases SP thymocytes. (**A**) Left: representative flow plots of CD4^+^ and CD8^+^ on reconstituted thymocytes in bone marrow chimeras. Right: percentages of CD4^+^ SP and CD8^+^ SP thymocytes among the reconstituted thymocytes (WT→WT, *n* = 6; WT→*Copa^E241K/+^*, *n* = 6; WT→*Sting^gt/gt^*, *n* = 5; WT→*Copa^E241K/+^*/*Sting^gt/gt^*, *n* = 5). (**B**) Left: representative flow analysis of CD69 and TCR-β on reconstituted thymocytes in bone marrow chimeras. Right: percentages of CD69^hi^ TCRβ^hi^ and CD69^lo^ TCRβ^hi^ among the reconstituted thymocytes. (**C**) Percentages of CD69^lo^ MHC-II^hi^ (mature stage 2) among the reconstituted CD4^+^ and CD8^+^ SP thymocytes. Flow gating strategy is in Supplemental Figure 5A. WT→WT, *n* = 7; WT→*Copa^E241K/+^*, *n* = 6; *Copa^E241K/+^*→WT, *n* = 6; *Copa^E241K/+^*→*Copa^E241K/+^*, *n* = 4; WT→*Sting^gt/gt^*, *n* = 4; WT→*Copa^E241K/+^*/*Sting^gt/gt^*, *n* = 3. Data in **A**–**C** were pooled from at least 2 independent experiments and are mean ± SD. Two-way ANOVA with Šidák’s multiple-comparison test was used for statistical analysis in **A** and **B**. One-way ANOVA and Bonferroni’s multiple-comparison test were used in **C**. A *P* value of less than 0.05 was considered statistically significant.

Having found that STING is activated in the thymic epithelium of *Copa^E241K/+^* mice, we next wanted to assess how it influenced thymocyte development. As in our previous study (5), we observed that *Copa^E241K/+^* mice have an increase in single positive (SP) thymocytes (Supplemental Figure 4, A and C) and postselection thymocytes (Supplemental Figure 4B). To assess whether activated STING triggered these changes, we examined STING-deficient *Copa^E241K/+^* mice (*Copa^E241/+^/Sting1^gt/gt^* mice) and found that the increase in the thymocyte populations was completely reversed (Supplemental Figure 4, A–C). We evaluated thymic APCs such as B cells and conventional dendritic cells (cDCs) (Supplemental Figure 2, A and B) and found that although STING activation does not affect the total cell counts of these APCs (Supplemental Figure 2C), it enhanced the maturation of B cells in *Copa^E241K/+^* mice (Supplemental Figure 2D). We next used bone marrow chimeras to determine whether activated STING in the radioresistant stromal compartment or the hematopoietic compartment caused the changes to the thymocyte populations. We transplanted WT bone marrow into irradiated *Copa^E241K/+^* mice and confirmed *Copa^E241K/+^* thymic stroma induced increased SP cells (Figure 2A and Supplemental Figure 5A) similar to unmanipulated *Copa^E241K/+^* mice. In contrast, the alterations to the thymocyte populations were reversed when WT bone marrow was transplanted into irradiated *Copa^E241/+^/Sting1^gt/gt^* mice (Figure 2, A and B), suggesting that STING exerts its influence on thymocyte populations from within the thymic stroma.

Our prior work indicated that higher type I IFN levels in TECs of *Copa^E241K/+^* mice imprint thymocytes with elevated interferon stimulated genes (ISGs) and promote an expansion of late-stage SP cells (2). By analyzing the SP thymocytes in reciprocal bone marrow chimera, we confirmed that the upregulated ISGs in *Copa^E241K/+^* SP thymocytes are largely due to STING activation in the stroma instead of hematopoietic cells (Supplemental Figure 5B). We then used a flow cytometry staining strategy that subsets increasingly mature SP cells into semimature, mature 1, and mature 2 populations (19) (Supplemental Figure 5, A, C, and D). Consistent with the results from above, we found that loss of STING in the thymic stroma reversed the expansion of M2 cells in the *Copa^E241K/+^* thymus (Figure 2C and Supplemental Figure 5D). Taken together, these data establish that chronic STING activation in *Copa^E241K/+^* TECs imprints SP thymocytes with increased ISGs, results in an increase in postselection SP thymocytes, and promotes the expansion of late-stage SP thymocytes.

To assess whether chronic STING activation within T cell progenitors contributes to any of the alterations observed, we performed mixed bone marrow chimeras in which we evenly mixed *Copa^E241K/+^* and WT bone marrows for transfer into lethally irradiated hosts (Supplemental Figure 6A). When we analyzed the reconstituted thymus, we found no differences in SP expansion or maturation between donors within the same host (Supplemental Figure 6, D–G). However, the reduction of *Copa^E241K/+^* -derived thymocytes when competing with WT cells within the same host reflected a compromised ability in their survival, proliferation, or both (Supplemental Figure 6, B and C). This defect in the hematopoietic compartment of *Copa^E241K/+^* mice brought out by the mixed chimeras reminded us of the pro-apoptotic effects of STING activation on immune cells and cytopenia in the SAVI mouse models (12, 20) and suggested that *Copa^E241K/+^* mice might also be prone to immune deficiencies. Despite the similarities between COPA syndrome and SAVI, the largely normal cellularity of immune cells and the generally milder lung disease in the *Copa^E241K/+^* mouse model (5) suggest that STING signaling in these 2 disorders might be distinct.

To determine to what extent STING-mediated type I IFN signaling contributes to altered thymocyte development, we next transferred bone marrow from the interferon (alpha and beta) receptor (Ifnar) knockout (*Ifnar^–/–^*) mice into irradiated WT or *Copa^E241K/+^* hosts. Loss of IFNAR in hematopoietic cells completely blunted the upregulation of ISGs in thymocytes caused by the *Copa^E241K/+^* thymic stroma as well as the activation of thymic B cells (Supplemental Figure 7A and Supplemental Figure 8). However, the expansion of postselection SP thymocytes was not fully rescued by IFNAR deficiency (Supplemental Figure 7, B–E), suggesting that additional mechanisms that map specifically to the thymic stroma may be involved. Although a growing body of work indicates transient STING activation induces noncanonical autophagy (21, 22), the role of STING on autophagic function in TECs has not been explored. Based on observations in prior work (1, 5), we speculated that chronic STING activation in TECs might actually impair macroautophagy (hereafter autophagy), a cellular process critical for processing and presenting self-peptides to thymocytes, particularly during negative selection (23). We reasoned that if persistent STING activation in *Copa^E241K/+^* mice did indeed disrupt autophagic function in TECs, then this might account for the increase in postselection SP thymocytes that persisted despite loss of IFNAR on hematopoietic cells.

### Chronic STING signaling in TECs impairs autophagy.

**Figure 3:**
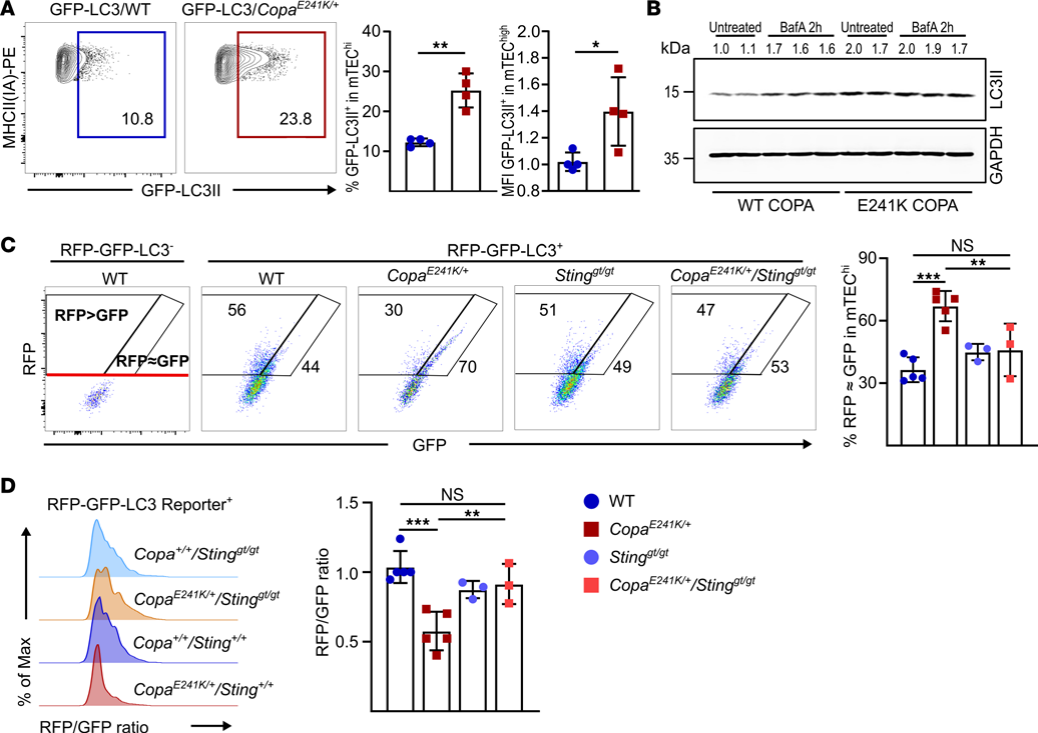
Constitutive activation of STING in the thymus impairs autophagic flux. (**A**) Left: flow analysis of autophagosome-associated LC3 (LC3II) in mTEC^hi^ from GFP-LC3 and GFP-LC3 × *Copa^E241K/+^* mice. Right: percentage of LC3II-GFP^+^ population among total mTEC^hi^ and GFP median fluorescence intensity in mTEC^hi^ (GFP-LC3 × WT, *n* = 4; GFP-LC3 × *Copa^E241K/+^*, *n* = 4). Data were pooled from 2 independent experiments. (**B**) Representative immunoblot and densitometric analysis of LC3II following transient transfection of WT or E241K COPA-expressing plasmid into HEK293T cells that stably express STING. Data represent 3 independent experiments. (**C**) Quantitation of autophagic flux in mTECs of CAG-RFP-GFP-LC3 tandem reporter mice. Left: flow cytometry of autophagosome-associated (RFP≈GFP) and autolysosome-associated (GFP<RFP) LC3 in mTECs. Right: percentage of mTEC^hi^ with reduced autophagic flux (RFP-GFP-LC3 × WT, *n* = 5; RFP-GFP-LC3 × *Copa^E241K/+^*, *n* = 5; RFP-GFP-LC3 × WT × *Sting^gt/gt^*, *n* = 3; RFP-GFP-LC3 × *Copa^E241K/+^* × *Sting^gt/gt^*, *n* = 3). (**D**) Left: ratio of RFP/GFP fluorescence histogram in mTECs expressing LC3 tandem reporter. Right: mean RFP/GFP ratio in mTECs (RFP-GFP-LC3 × WT, *n* = 5; RFP-GFP-LC3 × *Copa^E241K/+^*, *n* = 5; RFP-GFP-LC3 × WT × *Sting^gt/gt^*, *n* = 3; RFP-GFP-LC3 × *Copa^E241K/+^* × *Sting^gt/gt^*, *n* = 3). Data in **C** and **D** were pooled from 3 independent experiments and are presented as mean ± SD. Unpaired, parametric, 2-tailed Student’s *t* test was used for statistical analysis in **A**. One-way ANOVA with Bonferroni’s multiple-comparison test was used in **C** and **D**. A *P* value of less than 0.05 was considered statistically significant.

To investigate the role of chronic STING activation on autophagy in our model, we crossed *Copa^E241K/+^* mice to GFP-LC3 reporter mice (24) and measured GFP-LC3II^+^ by flow cytometry in TECs. Interestingly, mTEC^hi^ cells in *Copa^E241K/+^* mice had a striking increase in the percentage of cells expressing autophagosome-associated GFP-LC3II (Figure 3A). To determine whether the increase in autophagosomes reflected enhanced autophagy initiation or impaired autophagic flux, we expressed WT and E241K mutant COPA in HEK293T cells that stably express STING. We cultured the cells with and without bafilomycin A (BafA), which inhibits autophagosome/lysosome fusion (25). In cells expressing WT COPA, we observed an expected increase in LC3II levels after treatment with BafA. In cells expressing E241K mutant COPA, however, we observed higher levels of LC3II at baseline that did not increase in response to BafA treatment, consistent with impaired autophagic flux (Figure 3B).

To further dissect how STING activation might regulate autophagy in the thymic epithelium, we crossed *Copa^E241/+^* mice with *CAG-RFP-EGFP-LC3* tandem reporter mice so that we could quantitate autophagic flux in TECs by flow cytometry (26, 27). Because EGFP is more sensitive than RFP to the acidic environment of autolysosomes, cells with higher flux have less EGFP and, thus, an increased RFP/GFP ratio (Figure 3, C and D). Consistent with our data above, *Copa^E241/+^* mTEC^hi^ cells had a lower RFP/GFP ratio than WT mTEC^hi^ cells, indicating impaired autophagic flux. In addition, the RFP/GFP ratio returned to WT levels in STING-deficient *Copa^E241/+^/Sting1^gt/gt^* mice (Figure 3, C and D). Collectively, these findings indicate that chronic STING activation in mTEC^hi^ cells impairs autophagic flux, which is vital for delivery of endogenous self-peptides to developing T cells (23, 28).

### Chronic STING activation in TECs impairs T cell selection and alters the T cell repertoire.

Having found that chronic STING activation impaired autophagic flux in mTECs of *Copa^E241/+^* mice, we next wanted to examine the impact of this on repertoire selection of T cells. We employed the RiP-mOVA/OT-II system to study negative selection of T cells. RiP-mOVA mice express membrane-bound ovalbumin (mOVA) as a neo–self-antigen within mTECs and OT-II cells have a TCR specific for ovalbumin peptide. Normally, when OT-II cells traffic through the thymus and encounter ova peptide presented by major histocompatibility complex class II (MHC-II) on mTECs, they undergo clonal deletion and die (29).

**Figure 4:**
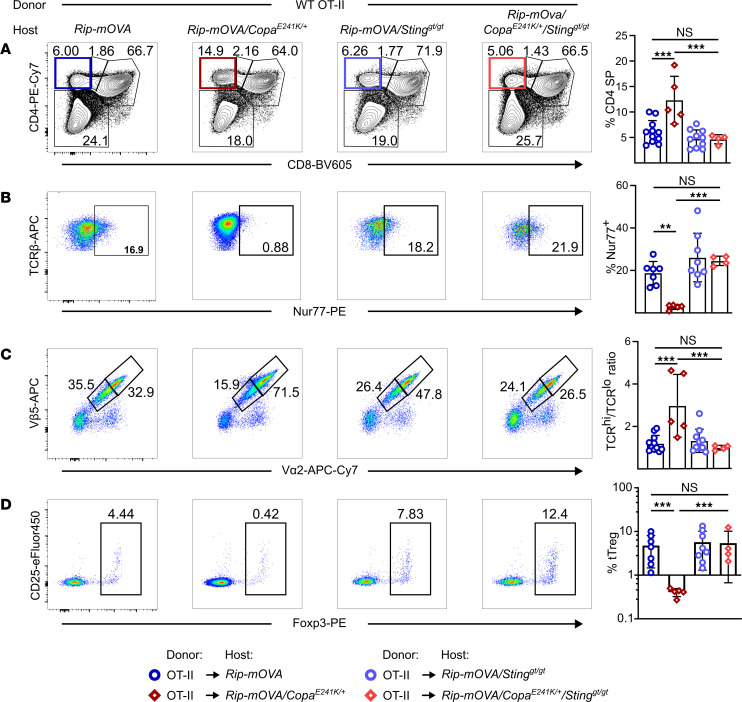
Activated STING impairs negative selection of T cells and alters the T cell repertoire. (**A**) Left: representative flow analysis of CD4^+^ and CD8^+^ on reconstituted thymocytes in bone marrow chimeras. Right: percentage of CD4^+^ SP thymocytes among the reconstituted thymocytes (OT-II→Rip-mOVA × WT, *n* = 11; OT-II→Rip-mOVA × *Copa^E241K/+^*, *n* = 5; WT→Rip-mOVA × WT × *Sting^gt/gt^*, *n* = 10; WT→Rip-mOVA × *Copa^E241K/+^* × *Sting^gt/gt^*, *n* = 4). (**B**) Left: flow analysis of TCR-β and Nur77 expression in the reconstituted CD4^+^ SP in the bone marrow chimeras. Right: percentage of Nur77^+^ population among CD4^+^ SP thymocytes (OT-II→Rip-mOVA × WT, *n* = 7; OT-II→Rip-mOVA × *Copa^E241K/+^*, *n* = 5; WT→Rip-mOVA × WT × *Sting^gt/gt^*, *n* = 8; WT→Rip-mOVA × *Copa^E241K/+^* × *Sting^gt/gt^*, *n* = 4). (**C**) Left: flow analysis of Vβ5 and Vα2 in CD4^+^ SP thymocytes in the bone marrow chimeras shown in (**A**). Right: ratio of TCR^hi^ versus TCR^lo^ among CD4^+^ SP thymocytes. (**D**) Left: flow analysis of CD25 and Foxp3 in CD4^+^ thymocytes in the bone marrow chimeras. Right: percentage of thymic Tregs among CD4^+^ thymocytes (OT-II→Rip-mOVA × WT, *n* = 7; OT-II→Rip-mOVA × *Copa^E241K/+^*, *n* = 5; WT→Rip-mOVA × WT × *Sting^gt/gt^*, *n* = 8; WT→Rip-mOVA × *Copa^E241K/+^* × *Sting^gt/gt^*, *n* = 4). Data were pooled from 3 independent experiments. Data are mean ± SD. One-way ANOVA and Bonferroni’s multiple-comparison test (log normal distribution in **D**) were used for statistical analysis. A *P* value of less than 0.05 was considered statistically significant. All host mice are on Rip-mOVA background.

We transplanted bone marrow from OT-II transgenic mice into RiP-mOVA mice bred onto the *Copa^E241K/+^*, *Copa^+/+^/Sting1^gt/gt^*, and *Copa^E241K/+^/Sting1^gt/gt^* genetic backgrounds and analyzed clonal deletion of OT-II thymocytes. In the *Copa^E241K/+^* background, we found a significant increase of OT-II CD4^+^ SP cells in the thymus compared with the WT background (Figure 4A). Remarkably, loss of STING in *Copa^E241K/+^/Sting1^gt/gt^* mice restored the percentages of OT-II CD4^+^ SP cells to levels comparable with WT mice (Figure 4A). We next evaluated clonotypic CD4^+^ SP cells for expression of cell surface CD5^+^ and intracellular Nur77, markers indicating the engagement and strength of TCR signaling (30). OT-II CD4^+^ SP cells from *Copa^E241/+^* mice failed to upregulate both CD5^+^ and Nur77 compared with SP thymocytes from WT and *Copa^E241K/+^/Sting1^gt/gt^* mice (Figure 4B and Supplemental Figure 9A). Moreover, the surface Vβ5 and Vα2 expression on OT-II CD4^+^ SP cells from *Copa^E241/+^* mice remained intact compared with CD4^+^ SP cells from WT and *Copa^E241K/+^/Sting1^gt/gt^* mice (Figure 4C), suggesting a lack of TCR engagement due to STING activation in thymic epithelium. Lastly, we found impaired generation of Tregs in *Copa^E241/+^* thymi (Figure 4D), which is also consistent with a defect in self-antigen–TCR interactions in the thymic medulla. Taken together, our data demonstrate that STING activation in the thymic epithelium perturbs negative selection, a key event in the establishment of central tolerance during T cell development, and thereby contributes to autoimmunity and disease in COPA syndrome and possibly other STING-associated immune diseases.

To determine whether the above defect could be applied more broadly, we first assessed the CD5^+^ level on polyclonal SP thymocytes and found that SP thymocytes in *Copa^E241K/+^* mice have a modest but significant drop in CD5^+^ on SP cells (Supplemental Figure 9B), suggesting that the selection of T cells across a broad range of specificities might be affected. Next, we compared TCRVβ chain profiles in WT, *Copa^E241K/+^*, and *Copa^E241K/+^/Sting1^gt/gt^* mice. Not surprisingly, the TCRVβ repertoire of CD4^+^ SP and CD8^+^ SP thymocytes in *Copa^E241K/+^* mice showed significant shifts that were rescued by STING deficiency (Supplemental Figure 9E). In contrast, the TCRVβ repertoire on DP thymocytes stayed largely normal compared with cells from WT and *Copa^E241K/+^/Sting1^gt/gt^* thymi (Supplemental Figure 9, C and D), suggesting that the alterations to the TCR repertoire on SP thymocytes occurred primarily in the *Copa^E241K/+^* medulla rather than the cortex. Interestingly, when we analyzed Vβ chains on DN thymocytes, we also observed signs of an altered TCR repertoire (Supplemental Figure 9, C and D). Given the ligand independence of pre-TCR activation during β-selection (31), we speculate this change could either result from the heightened IFNs in the medulla or a T cell–intrinsic response to STING activation.

To further examine how different thymic compartments help shape the SP TCR repertoire, we mixed congenically marked *Copa^E241K/+^* and WT bone marrows together and transferred them into lethally irradiated *Copa^E241K/+^* and *Copa^E241K/+^/Sting1^gt/gt^* hosts (Supplemental Figure 10A). We individually assessed representative Vβ chains and observed differences caused by the host stroma (Vβ5 on CD4^+^ and CD8^+^ SP, Supplemental Figure 10B), further corroborating our data showing that stromal STING activation influences the T cell repertoire. Meanwhile, we found a shift in the Vβs of WT donor cells mixed with *Copa^E241K/+^* bone marrow in *Copa^E241K/+^/Sting1^gt/gt^* hosts (Vβ13 on CD8^+^ SP, Supplemental Figure 10B), suggesting a potential influence of activated STING in thymic APCs. We also detected a shift in Vβ usage between donors within the same hosts (Vβ13 on CD4^+^ and CD8^+^ SP, Supplemental Figure 10B), indicating that T cell–intrinsic STING activation may be playing a role. Taken together, our findings suggest that chronic STING activation causes a defect in negative selection of T cells and an overall shift in the T cell repertoire.

### A systemic STING agonist increases autoreactive T cells in the thymus.

**Figure 5:**
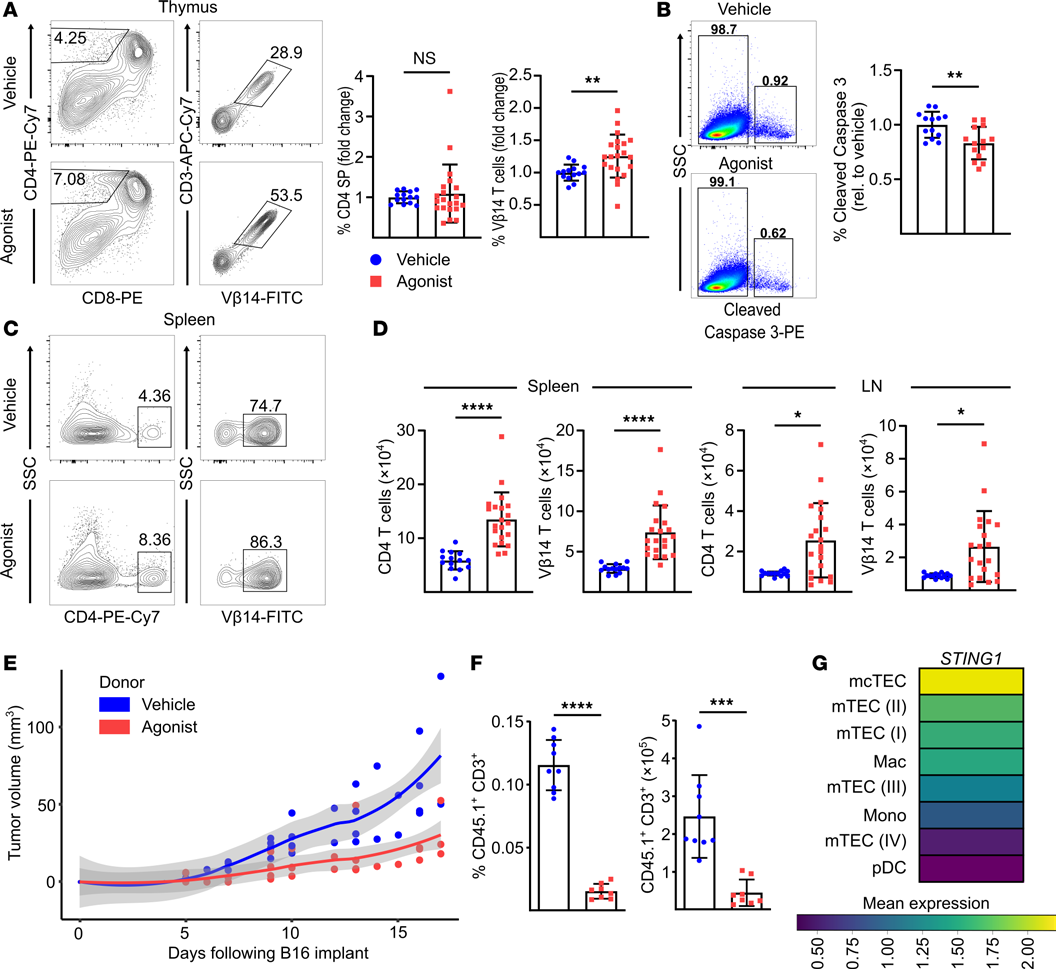
A systemic STING agonist increases autoreactive T cells in the thymus. (**A**) Left: CD4^+^ and CD8^+^ profile of thymocytes and CD3^+^ and Vβ14^+^ expression of CD4^+^ SP thymocytes in *Rag1^–/–^* *Tyrp1^B-w/wt^* TCR mice treated with diABZI STING agonist or vehicle. Right: change in percentage of total thymic CD4^+^ SP and Vβ14^+^CD4^+^ SP following STING agonist treatment (vehicle, *n* = 14; agonist, *n* = 21; compiled from 3 independent experiments). (**B**) Left: cleaved caspase 3 on CD5^hi^ TCRβ^hi^ thymocytes in vehicle- and agonist-treated mice. Right: percentage of thymocytes undergoing clonal deletion in agonist-treated mice relative to vehicle treatment (vehicle, *n* = 13; agonist, *n* = 14; compiled from 3 independent experiments). Unpaired, parametric, 2-tailed Student’s *t* test was used for statistical analysis. (**C**) Left: flow analysis of splenic CD4^+^ SP Vβ14^+^ autoreactive T cells in vehicle- and agonist-treated mice. Right: absolute number of CD4^+^ SP Vβ14^+^ autoreactive T cells. (**D**) Absolute number of splenic and inguinal CD4^+^ SP and Vβ14^+^ autoreactive T cells in vehicle- and agonist-treated mice (vehicle, *n* = 14; agonist, *n* = 21; 3 independent experiments). LN, lymph node. (**E**) B16 melanoma growth in *Rag1^–/–^* mice that received x-ray radiation, PD-1 antibody, and adoptively transferred splenocytes from *Rag1^–/–^* Tyrp1^B-w/wt^ TCR mice treated with STING agonist (*n* = 5) or vehicle (*n* = 4) (data represent 3 independent experiments). Locally estimated scatter plot smoothing with 95% confidence interval of B16 tumor growth over time. (**F**) Quantitation of CD3^+^ CD45.1 donor cells within the periphery after transfer into CD45.2 host and treatment with agonist (*n* = 8) or vehicle (*n* = 9). Data were pooled from 3 independent experiments. (**G**) Mean expression of STING1 transcript in select cell types in human thymus. Data are mean ± SD. Two-tailed Mann-Whitney *U* test was used for statistical analysis unless indicated above. A *P* value of less than 0.05 was considered statistically significant.

Having found that constitutive STING activation in the thymic epithelium alters thymocyte development and selection, we wondered if our findings might be applied more broadly outside the context of the COPA syndrome model. Specifically, we sought to examine whether activation of STING in the thymic stroma using a systemic STING agonist developed for clinical use (for instance, cancer immunotherapy) would phenocopy our findings and promote the output of autoreactive T cell clones. To study this, we turned to tyrosinase-related protein 1 (TRP-1) TCR Tg mice, an MHC-II–restricted mouse model in which CD4^+^ T cells recognize an epitope of the endogenous TRP-1 (32). TRP-1 is expressed as a melanocyte self-antigen in mTECs and also in melanoma tumors. Expression of TRP-1 in mTECs results in the deletion of TRP-1–specific T cells in the thymus and prevents their escape into peripheral lymphoid organs (18, 33). Because our data suggested that activation of STING in mTECs leads to a defect in negative selection, we set out to study whether systemic delivery of a STING agonist would disrupt processing and presentation of the TRP-1 self-antigen to developing thymocytes. To assess this, we treated TRP-1 TCR Tg mice (on a *Rag1^–/–^* background) with the recently reported dimeric amidobenzimidazole (diABZI) STING agonist (34) every other day for 2 weeks (Supplemental Figure 11A). After a brief washout period, we assessed the thymus for the selection of TRP-1–specific T cells. In mice treated with the diABZI STING agonist, we found a significant increase in the percentages of CD3^+^Vβ14^+^ T cells in comparison with control mice treated with vehicle alone (Figure 5A). An examination of CD3^+^Vβ14^+^ signaled thymocytes showed significantly lower levels of cleaved caspase-3 in mice receiving the STING agonist, consistent with a decrease in clonal deletion of antitumor T cells in treated mice (35) (Figure 5B).

We assessed peripheral lymphoid organs for the presence of CD3^+^Vβ14^+^ T cells. On peripheral CD4^+^ T cells, we found that STING agonists had no impact on PD-1 levels (Supplemental Figure 11B). In lymph nodes and spleen, there was a significant increase in CD3^+^Vβ14^+^ T cells that escaped negative selection in the thymus (Figure 5, C and D, and Supplemental Figure 11C). To confirm that these T cells were functionally competent, we next sought to determine whether the cells could mediate an antitumor immune response because although TRP-1 is a self-antigen, it is also expressed in B16 melanoma tumors. After the washout period, we isolated secondary lymphoid organs from TRP-1 TCR Tg mice treated with STING agonist or control vehicle to collect total CD3^+^Vβ14^+^ T cells. From each treated mouse, we adoptively transferred harvested cells into a *Rag1^–/–^* mouse implanted with B16 melanoma tumor. We observed marked reduced growth rate of tumors in hosts that received CD3^+^Vβ14^+^ T cells from STING agonist–treated mice in comparison with tumors in mice that received control cells (Figure 5E). To assess the possibility that diABZI STING agonist induces nonspecific proliferation of peripheral T cells, splenocytes from WT CD45.1 donors were transferred into WT CD45.2 hosts, and host mice were administered vehicle or STING agonist twice. Agonist-treated donor cells had significantly reduced numbers (Figure 5F) compared with vehicle-treated control cells, indicating an absence of nonspecific proliferation. Taken together, activation of STING in the thymus independent of COPA syndrome leads to the escape of self-antigen–specific T cells that otherwise normally undergo negative selection, findings relevant for other immunoregulatory disorders and patients receiving small molecule drugs to modulate STING signaling during cancer immunotherapy.

### STING is highly expressed in mTECs in humans.

Finally, although the *Copa^E241/+^* mouse model closely phenocopies COPA syndrome in patients and is a powerful model to study the disease (2, 5), we next wanted to directly determine the potential relevance of thymic STING in human tissue and assess the levels of STING transcript in human thymus. We analyzed single-cell RNA sequencing data from a human thymus cell atlas and found that STING was variably expressed depending on the specific cell type (36). STING was expressed in both thymocytes and TECs, consistent with our data and that of others (2, 11) showing an effect of STING within each of these cellular compartments. Compared with thymocytes, mTECs generally had higher mean expression of *STING* and a greater fraction of each cell type expressing the gene (Supplemental Figure 11D). Among stromal cells, STING was most highly expressed in the thymic epithelium and exhibited elevated expression in mTECs, including those directly involved in processing and presenting tissue antigens to developing T cells (37) (Figure 5G and Supplemental Figure 11E). Interestingly, STING transcript levels in human mTECs were higher than those found in thymic macrophages and more than double the levels of monocytes or plasmacytoid DCs (Figure 5G and Supplemental Figure 11E) (10).

## Discussion

This work establishes that STING has a functional role in the thymic epithelium that can impact thymocyte development and selection. We found that activation of thymic STING significantly upregulated type I IFN signaling and unexpectedly impaired autophagic flux and the processing and presentation of peptide antigens. The changes to the thymic epithelium mediated by activated STING altered thymocyte maturation and caused both a defect in negative selection and shift in the T cell repertoire (Supplemental Figure 12). Importantly, we made these observations not only in the presence of pathogenic *COPA* mutations that cause autoimmune disease, but also after administering a systemic STING agonist being trialed for cancer patients. Thus, our data have broad implications for any setting that activates thymic STING, including not only the clinical scenarios we studied, but also in patients with systemic infections or other more common autoimmune syndromes such as systemic lupus erythematosus (SLE). Furthermore, these findings are relevant throughout the lifespan, given the recent landmark study that showed the thymus modulates the risk of both cancer and autoimmunity in adults (38).

Interestingly, the thymus is a common target organ in infectious diseases (39). Different viruses, including SARS-CoV-2, have been shown to directly target the thymic microenvironment and infect cells involved in thymocyte selection, including TECs (40, 41). Although we have not yet shown it directly, our work suggests that systemic viral or bacterial infections have the potential to activate thymic STING and alter T cell selection, which could in theory trigger autoimmune disease. Indeed, investigators have shown that viruses may disrupt central tolerance and promote autoimmunity by reducing TEC numbers or the expression of tissue-specific antigens (42). The activation of STING (and possibly other nucleic acid sensing molecules) in the thymus demonstrates another mechanism by which activation of an innate immune signaling pathway leads to loss of self-tolerance.

The major STING signaling output in TECs we identified was type I IFNs. There has been increasing interest in determining how IFNs in the thymus affect developing T cells, especially after it was shown that TECs have high levels of constitutive *IFNB* expression under homeostatic conditions (43). Further investigation is needed to understand how IFN secretion in the thymus is regulated, particularly given recent studies that suggest loss of IFN expression in the thymus may lead to the generation of IFN autoantibodies that predispose to severe COVID-19 and other infections (44). In addition to nucleic acid sensing molecules such as STING, the transcriptional activator AIRE (autoimmune regulator) may have a role in controlling IFN expression in TECs (45).

Thymic IFNs mediate the maturation of late-stage thymocytes and other cell types such as B cells and cDCs (19, 46, 47), which participate in shaping the TCR repertoire under physiological conditions. We detected enhanced maturation of SP thymocytes and B cells (Figure 2, Supplemental Figure 2D, and Supplemental Figure 5) when IFN production was augmented by activated STING in mTECs of *Copa^E241K/+^* mice. Loss of IFNAR in the hematopoietic compartment fully restored the B cell phenotype in the thymus and partially rescued SP thymocyte maturation and expansion (Supplemental Figures 7 and 8). These data support a model in which elevated IFNs in the mTEC compartment shift the T cell repertoire in the thymus to promote a loss of T cell tolerance and autoimmunity. We also identified impaired autophagic flux as another cellular response downstream of chronic STING activation in mTECs (Figure 3). Previous studies have shown that endogenous proteins gain access to the MHC-II presentation pathway within cells by autophagy (48). Moreover, macroautophagy serves as a unique pathway for tissue-specific antigen presentation in TECs, including mTECs and cortical thymic epithelial cells (cTECs) (23), thereby contributing to T cell selection and tolerance. In this study, we observed a defect in negative selection of autoreactive T cells using 2 types of TCR transgenic models (Figures 4 and 5). Diminished expression of Nur77 and CD5^+^ by autoreactive CD4^+^ SP cells in *Copa^E241K/+^* host (Figure 4B and Supplemental Figure 9A) suggests a lack of TCR engagement due to impaired self-antigen presentation. However, further work must be performed to further characterize the importance of impaired autophagy to a loss of tolerance in our model as well as the contribution that the pro-apoptotic effect of STING activation has on mTECs and T cell selection (Figure 1E).

Although our study identified a functional role of STING activation in mTECs, the role of STING in cTECs remains unexplored. Using immunofluorescence staining, we primarily detected pSTING within the thymic medulla area, suggesting that STING activation was more prominent in mTECs instead of cTECs. In support of this, our assessment of several Vβs on DP thymocytes in WT, *Copa^E241K/+^*, and *Copa^E241K/+^Sting^gt/gt^* mice did not reveal a significant change to positive selection given the normal Vβ usage by DP thymocytes in *Copa^E241K/+^* mice (Supplemental Figure 9D). This observation aligns with previous work in which we studied 3 different TCR transgenic mice that showed normal positive selection in the *Copa^E241K/+^* mouse background (5), suggesting that cTECs may not be significantly altered in our model. The increased apoptotic rate of DP thymocytes (Supplemental Figure 3B) in *Copa^E241K/+^* mice might reflect either a cell autonomous role of STING activation causing death of DP cells or a defect in cTEC antigen presentation that results in more “death by neglect.” Nevertheless, based on the functional readouts we employed to study thymic repertoire development, we think the extent of STING activation in cTECs is limited in our model, and further investigation is warranted.

The effects of T cell–intrinsic STING activation appear to be pleiotropic and can promote the generation of stem-like memory T cells (8), skew T cells toward specific helper cell subsets (11), and cause death (13, 17, 49), including in developing thymocytes (15). In addition to mTECs, we observed increased apoptosis of DP thymocytes (Supplemental Figure 3B). Both cell types are highly proliferative and undergo fast turnover (50, 51), suggesting that activated STING might synergize with stress signals associated with proliferation to trigger cell death. The compromised ability of *Copa^E241K/+^* bone marrow to reconstitute thymi in the mixed bone marrow chimeras (Supplemental Figure 6, B and C) in comparison with WT cells also suggests that activation of STING may be influencing the survival of progenitor cells before they populate the thymus. Immune cell death in the context of STING activation has been mapped to diverse biological events, including ER stress and intrinsic mitochondrial apoptosis (13, 52), all of which may influence cell survival. It is noteworthy, however, that COPA syndrome patients do not manifest immunodeficiency (1) and the cellularity of immune cells in *Copa^E241K/+^* mice (5) are largely normal compared with SAVI mice, which are immunodeficient (12, 14, 53).

There has been an intense interest to modulate STING signaling therapeutically given its central role in a broad range of biological contexts important to human health and disease (54). Our ability to activate STING in the thymus with small molecules and alter T cell selection is highly relevant for cancer treatment protocols that employ these drugs (55). We showed not only how STING agonists can enhance antitumor T cell responses, but also pinpointed a potential mechanism by which STING agonists might provoke autoimmune reactions. When administered with checkpoint inhibitors, STING agonists acting on the thymus may predispose to immune-related adverse events (56). In any case, we demonstrate the critical importance of studying off-target effects of systemically administered STING therapeutics (57), and our discovery of STING’s functional role in the thymus will be similarly important in clinical contexts that trial STING inhibitors. Additional modulation of T cell activation versus apoptosis via STING agonist is likely to be dose dependent. The dose we chose had an impact on T cell survival, as demonstrated by the reduction in transferred T cell numbers and viability. Exploring a larger range of doses will enable identification of an optimal dose that does not induce apoptosis but can activate T cells against specific targets like cancer cells (58).

Our study has limitations. Persistent activation of thymic STING led to an expected increase in type I IFNs, but it remains unclear why macroautophagic flux was impaired. Activation of STING has been shown to induce noncanonical autophagy (21), although this degradation pathway is not known to have a role in antigen processing and presentation. Further study is needed to understand how persistent STING activation impacts STING-induced autophagy and whether it directly affects macroautophagic function in TECs. Although we show a correlation between impaired autophagic flux and defective negative selection in *Copa^E241K/+^* mice, at present our data definitively establish that the changes to autophagy in TECs directly alter thymocyte selection. Other questions requiring further exploration include understanding whether type III IFNs are involved in mediating thymic selection in our system. An analysis of systemic STING agonists on other stromal (e.g., cTEC) and hematopoietic populations in the thymic environment is also needed.

In conclusion, we uncovered an unexpected role for STING in TEC function and provide new insight into how STING shapes the T cell repertoire to contribute to autoimmunity and immune dysregulation. These findings have important implications for any setting in which systemic inflammation activates STING in the thymic stroma. This includes not only COPA syndrome, but potentially other autoimmune diseases such as SLE. In addition, our findings have relevance for STING agonist use in cancer treatment or understanding how T cell responses might be altered during systemic infections that activate thymic STING.

## Methods

### Sex as a biological variable.

Our study examined male and female animals, and similar findings are reported for both sexes.

### Mice.

*Copa^E241K/wt^* mice were previously generated in our laboratory (5). The B6.SJL-*Ptprc^a^ Pepc^b^*/BoyJ (B6 CD45.1), B6(Cg)-Ifnar1^tm1.2Ees^/J (*Ifnar^–/–^*), B6.Cg-Tg(Tcra,Tcrb)425Cbn/J (OT-II), C57BL/6J-Sting1^gt^/J, C57BL/6-Tg(CAG-RFP/EGFP/Map1lc3b)1Hill/J (CAG-RFP-EGFP-LC3), B6.129S7-Rag1^tm1Mom^/J (*Rag1^–/–^*), and B6.Cg-Rag1^tm1Mom^ Tyrp1^B-w^ Tg(Tcra,Tcrb)9Rest/J *(Rag1^–/–^* *Tyrp1^B-w/wt^* TCR) mice were acquired from The Jackson Laboratory. GFP-LC3 transgenic mice (24) were provided by Jayanta Debnath at UCSF. All mice were housed in a specific pathogen-free facility at UCSF.

### Histology and confocal microscopy.

Isolated thymi were fixed for 1 h in PBS with 4% paraformaldehyde (PFA), cryopreserved overnight in 30% sucrose, embedded in disposable base molds (VWR) with Tissue-Tek OCT (Sakura Finetek USA), frozen sectioned at 10 μm, and placed on Superfrost Plus slides (Thermo Fisher Scientific). Tissue sections were blocked with goat serum (Sigma) and then stained with antibodies against pSTING (D8F4W; Cell Signaling Technology), cytokeratin 5 (EP1601Y; Abcam), and cytokeratin 8 (EP1628Y; Abcam). Alexa Fluor 488–conjugated goat anti-rabbit secondary antibody (A-11008) was acquired from Thermo Fisher Scientific, and sections were counterstained with DAPI (BioLegend). Images were captured with a Nikon Crest LFOV Spinning Disk/C2 confocal microscope.

### TEC isolation.

For flow analysis, TECs were isolated as previously described (2). In brief, thymi from 4-week-old mice were minced with razor blades on ice and digested 3 times at 37°C (DMEM high glucose [Cytiva] with 2% FBS [Atlas Biologicals], 100 μg/mL DNase I [Worthington Biochemical], and 100 μg/mL Liberase TM [Roche]) until no tissue fragments remained. Single cells harvested from each digestion were quenched, washed with magnetic activated cell sorting buffer (PBS with 5 mg/mL BSA and 2 mM EDTA), and merged together for the next step. Total cells from 1 thymus were applied to a discontinuous Percoll PLUS (Cytiva) gradient including densities of 1.115 (4 mL), 1.065 (2 mL), and 1.0 (2 mL). The gradient was centrifuged at 800*g* (4°C) for 30 min. TECs were then extracted from the interphase of 1.065 and 1.0 densities and further analyzed by flow cytometry.

For Western blot analysis, thymi were first digested at 37°C (DMEM high glucose with 2% FBS, 100 μg/mL DNase I, and 100 μg/mL Liberase TM [Roche]) and then washed with magnetic activated cell sorting buffer (PBS with 5 mg/mL BSA and 2 mM EDTA). Thymocytes, hematopoietic cells, and endothelial cells were depleted with biotinylated antibody cocktail (BioLegend: α-CD45 [30-F11], α-CD4 [GK1.5], α-CD8 [53-6.7], α-CD11c [N418], α-CD11b [M1/70], and α-CD31 [390]) plus streptavidin-Dynabeads (65601; Thermo Fisher Scientific). The purity of the mTECs after the antibody depletion was tested by flow analysis with an average yield above 80% epithelial cell adhesion molecule (EpCAM) positive. Lysates of 50,000 TEC cells were loaded for each lane in the Western blot.

### Thymic B cell and DC preparation.

Thymi were minced and digested at 37°C (DMEM high glucose with 2% FBS, 100 μg/mL DNase I, and 1 mg/mL collagenase D [Roche]) for 30 min. Single-cell suspension was filtered through 64 μm cell strainers and pelleted by centrifugation at 700*g* × 7 min. B cells and cDCs were either directly analyzed by flow or enriched with biotinylated antibody cocktail (α-CD4 and α-CD8) plus streptavidin-Dynabeads for further analysis.

### RNA sequencing and data analysis.

RNA sequencing data of murine TECs was previously generated (2). In brief, cDNA was generated from isolated RNA with the Nugen Ovation method (7102-A01; Tecan), and a sequencing library was created with the Nextera XT method (FC-131-1096; Illumina). Libraries were sequenced on an Illumina HiSeq 4000, and resulting reads were aligned to Ensembl build GRCm38.78 with STAR (v2.4.2a). Read counts per gene were inputted to DESeq2 (v1.26.0), and the resulting differential gene expression profile was plotted with EnhancedVolcano (v1.18.0).

Human *STING1* transcript expression was assessed with a publicly available cell atlas of human thymus (36) (https://doi.org/10.5281/zenodo.5500511) and Python toolkits Scanpy (v1.9.4), pandas (v2.0.3), and NumPy (v1.25.0).

### Flow cytometry and antibodies.

Single-cell suspensions of thymocytes and splenocytes were prepared by mechanically disrupting the thymus and spleen and passing the cells through 64 μm filters (Genesee Scientific). Following red blood cell lysis (BioLegend), cells were maintained on ice in RPMI 1640 supplemented with 5% FBS. An aliquot of each cell suspension was mixed with AccuCheck Counting Beads (PCB100; Invitrogen) and analyzed by flow cytometry, and total cell number was calculated according to the manufacturer’s protocol.

For evaluation of surface markers, cells were blocked with 10 μg/mL anti-CD16/32 for 15 min at room temperature and then stained with indicated antibodies in FACS buffer (PBS with 2% BSA) on ice for 1 h.

For flow analysis of intracellular cytokines in TECs, freshly isolated TECs from Percoll gradient were washed in FACS buffer (PBS with 2% BSA) and then stained with anti-CD16/32 followed by flow antibodies toward surface markers. The cells were washed and fixed with 2% PFA in PBS for 30 min at room temperature and then washed with FACS buffer (PBS with 2% BSA). The cells were lastly stained with antibodies against intracellular cytokines in the presence of permeabilization buffer (eBioscience Foxp3/Transcription Factor Staining Buffer Set) overnight at 4°C. The cells were washed with permeabilization buffer and analyzed by flow cytometry the next morning.

For apoptosis measurement, freshly isolated thymic cells were surface stained with flow antibodies, washed, and incubated with annexin V and 7-AAD (BioLegend) at room temperature for 15 min before acquisition on a flow machine.

To quantify cellular senescence, freshly isolated thymic cells were cultured with C12FDG and BafA in RPMI 1640 (10% FBS and 1× GlutaMAX (Gibco) in a tissue culture incubator for 30 min and then stained with flow antibodies.

Flow cytometry data were acquired on a FACSVerse, FACSAria Fusion, or LSRFortessa system (BD Biosciences) and analyzed with FlowJo v10.

Antibodies to the following were purchased from BD Biosciences: mouse TCR Vβ screening panel (557004), Vβ12^+^ (MR11-1), and Vβ14^+^ (14-2); BioLegend: annexin V Apoptosis Detection Kit with 7-AAD (640922), CCL5 (2E9/CCL5), CD3^+^ (145-2C11), CD5^+^ (53-7.3), CD19 (6D5), CD25 (PC61), CD45 (30-F11), CD62L (MEL-14), CD80 (16-10A1), EpCAM (G8.8), H-2 MHC class I (M1/42), I-A^b^ MHC class II (AF6-120.1), IFN-γ (XMG1.2), IL-6 (MP5-20F3), Ly-6C (HK1.4), Ly-51 (6C3), NK1.1 (PK136), TCR-γ/δ (GL3), TNF-α (MP6-XT22), Vα2 (B20.1), Vβ5.1/2 (MR9-4), Vβ6 (RR4-7), Vβ8.1/2 (MR5-2), Vβ8.3 (1B3.3), and Vβ13 (MR12-4); Cell Signaling Technology: cleaved caspase 3 (D3E9); Cytek Biosciences: B220 (RA3-6B2), CD4^+^ (RM4-5), CD8α (53-6.7), CD11c (N418), CD44 (IM7), CD45.2 (104), CD69 (H1.2F3), F4/80 (BM8.1), Gr1 (RB6-8C5), and TCR-β (H57-597); Thermo Fisher Scientific: CD45.1 (A20), CD69 (H1.2F3), and Nur77 (12.14); and UCSF’s Hybridoma Core Facility: CD16/CD32 (2.4G2). SYTOX Red dead cell stain was purchased from Thermo Fisher Scientific.

### Quantitative real-time PCR.

RNA was isolated with the PicoPure RNA isolation kit (Thermo Fisher Scientific) and reverse transcribed to cDNA with SuperScript III and oligo-dT_16_ primers (Thermo Fisher Scientific). Quantitative PCR was performed on a Bio-Rad CFX thermal cycler with TaqMan Fast Advanced Master Mix and TaqMan Gene Expression assays from Thermo Fisher Scientific: *Gapdh*, Mm99999915_g1; *Ifit1*, Mm00515153_m1; *Isg15*, Mm01705338_s1; *Fezf2*, Mm01320619_m1; *Aire*, Mm00477461_m1.

### Immunoblotting and antibodies.

Cells were lysed in Cold Spring Harbor NP-40 lysis buffer (150 mM NaCl, 50 mM Tris, pH 8.0, and 1.0% Nonidet P-40) containing protease and phosphatase inhibitors (PMSF, NaF, Na_3_VO_4_, and Roche PhosSTOP). Lysates were cleared by centrifuging at 10,000*g* for 10 min at 4°C, boiled at 95°C for 5 min with SDS loading dye containing β-mercaptoethanol, size separated on SDS-PAGE gels, and wet transferred onto PVDF membrane. Membranes were blocked in TBS with 0.05% Tween 20 (TBS-T) and 5% nonfat dry milk for 1 h at room temperature, followed by overnight incubation at 4°C with primary antibodies diluted 1:5,000 in TBS-T with 5% BSA. Membranes were washed 3 times with TBS-T for 10 min, incubated with 1:20,000 diluted HRP-conjugated IgG secondary antibody (Jackson Immunoresearch) for 1 h at room temperature, and washed 3 times with TBS-T for 10 min followed by once with TBS for 10 min. Lastly, bands were visualized with SuperSignal West Femto Chemiluminescent Substrate (Thermo Fisher Scientific) and Bio-Rad’s ChemiDoc MP imager.

Antibodies to the following were purchased from Cell Signaling Technology: LC3B (D11) and pSTING (D8F4W); and Santa Cruz Biotechnology: GAPDH (6C5).

### Plasmids and transient transfection.

FLAG-tagged WT and E241K human COPA expression plasmids were previously described (1). EGFP-tagged human STING lentiviral plasmid was a gift from Tomohiko Taguchi (Tohoku University, Sendai, Japan). HEK293T cells (ATCC) were seeded at 40%–60% density, transiently transfected with jetOPTIMUS reagent (Polyplus) per the manufacturer’s protocol, and lysed 48 h later for immunoblotting.

### Bone marrow chimeras.

Host mice aged 3–4 months were fasted overnight and then depleted of bone marrow with 2 doses (550 rads each, separated 12 h apart) of x-ray radiation (X-Rad320; Precision X-Ray Irradiation). Twenty-five–gauge needles mounted on syringes filled with RPMI 1640 and 5% FBS were used to flush donor bone marrow cells from femurs and tibias of 8-week-old, gender-matched donor mice. T cells were depleted with CD90.2 antibody (30-H112; BioLegend) and streptavidin-Dynabeads. Ten million bone marrow cells suspended in 200 μL of saline were intravenously transferred into each host. Chimeras were housed under specific pathogen-free conditions and analyzed 6 to 8 weeks after reconstitution.

### In vivo STING activation.

Beginning at 5–7 weeks of age, STING agonist diABZI compound 3 (ProbeChem) was i.p. administered (0.25 mg/kg) to *Rag1^–/–^ Tyrp1^B-w/wt^* TRP-1 TCR mice every other day, 7 times, followed by 7 days of rest. Mice were then euthanized to harvest cells from thymi and peripheral lymphoid organs for analysis or adoptive transfer.

### Tumor challenge.

*Copa^E241K/wt^* and WT littermates were subcutaneously challenged with 1 × 10^5^ B16-F10 melanocytes (a gift from Lawrence Fong, UCSF) at the left flank. Tumor volume was estimated as length × width^2^/2, and mice were sacrificed at a volume of 1,000 mm^3^ (counted as death for Kaplan-Meier survival analysis).

For adoptive transfers, *Rag1^–/–^* mice were subcutaneously challenged with 5 × 10^4^ B16-F10 cells at the left flank on day 0. Mice received 500 rads of x-ray radiation and 125 μg of PD-1 antibody (CD279; BioXCell) i.p. on day 2. Total splenocytes from 1 *Rag1^–/–^ Tyrp1^B-w/wt^* TCR mouse given STING agonist or vehicle, as described above, were transferred into 1 tumor challenged *Rag1^–/–^* mouse on day 2. Mice received an additional 125 μg of PD-1 antibody i.p. every 4 days. Mice were euthanized 16 to 17 days following tumor inoculations unless tumor size necessitated earlier sacrifice.

### Adoptive transfer.

B6 CD45.1 donors were sacrificed and their spleens collected, and the splenocytes were isolated by teasing apart the tissue and passing the cell suspension through 64 μm filters. Splenocytes were resuspended at 50 million cells per milliliter of saline. Five hundred microliters of labeled splenocyte suspension was i.p. injected into WT CD45.2 hosts. Three hours after cell transfer, hosts were i.p. injected diABZI compound 3 (0.25 mg/kg) or vehicle. Two days after cell transfer, agonist or vehicle injection was repeated. On day 3, hosts were sacrificed and spleens collected. Splenocytes were isolated and surface stained for flow cytometry analysis as described above.

### Statistics.

Data are presented relative to the mean of WT or agonist treated mice. Statistical analysis was performed with Prism 9 (GraphPad) or R 4.0.0 (R Foundation for Statistical Computing). Unpaired, parametric, 2-tailed Student’s *t* test, 2-tailed Mann-Whitney *U* test, 1-way ANOVA with Bonferroni’s multiple-comparison test, or 2-way ANOVA with Šidák’s multiple-comparison test was used where indicated. A *P* value of less than 0.05 was considered statistically significant.

### Study approval.

All animal protocols were approved by UCSF’s IACUC.

### Data availability.

The RNA sequencing data are available at the Gene Expression Omnibus (accession number GSE153822). Values for data presented in Supplemental Figures 1–12 are provided in the Supporting Data Values file.

## Author contributions

AKS conceived and supervised the study, acquired funding, and reviewed and edited the article. ZD, CSL, and AKS designed the methods. ZD, CSL, and SK performed investigations. ZD, CSL, SK, and NS performed visualization. ZD and AKS contributed project administration. ZD and AKS wrote the original draft.

## Supplementary Material

Supplemental data

Unedited blot and gel images

Supporting data values

## Version 1 06/26/2025

In-Press Preview

## Version 2 09/02/2025

Electronic publication

## References

[1] Watkin LB (2015). COPA mutations impair ER-Golgi transport and cause hereditary autoimmune-mediated lung disease and arthritis. Nat Genet.

[2] Deng Z (2020). A defect in COPI-mediated transport of STING causes immune dysregulation in COPA syndrome. J Exp Med.

[3] Mukai K (2021). Homeostatic regulation of STING by retrograde membrane traffic to the ER. Nat Commun.

[4] Lepelley A (2020). Mutations in COPA lead to abnormal trafficking of STING to the Golgi and interferon signaling. J Exp Med.

[5] Deng Z (2020). A defect in thymic tolerance causes T cell-mediated autoimmunity in a murine model of COPA syndrome. J Immunol.

[6] Gall A (2012). Autoimmunity initiates in nonhematopoietic cells and progresses via lymphocytes in an interferon-dependent autoimmune disease. Immunity.

[7] Luksch H (2019). STING-associated lung disease in mice relies on T cells but not type I interferon. J Allergy Clin Immunol.

[8] Li W (2020). cGAS-STING–mediated DNA sensing maintains CD8^+^ T cell stemness and promotes antitumor T cell therapy. Sci Transl Med.

[9] Gutjahr A (2019). The STING ligand cGAMP potentiates the efficacy of vaccine-induced CD8+ T cells. JCI Insight.

[10] Jneid B (2023). Selective STING stimulation in dendritic cells primes antitumor T cell responses. Sci Immunol.

[11] Benoit-Lizon I (2022). CD4 T cell-intrinsic STING signaling controls the differentiation and effector functions of T_H_1 and T_H_9 cells. J Immunother Cancer.

[12] Bouis D (2019). Severe combined immunodeficiency in stimulator of interferon genes (STING) V154M/wild-type mice. J Allergy Clin Immunol.

[13] Wu J (2019). STING-mediated disruption of calcium homeostasis chronically activates ER stress and primes T cell death. J Exp Med.

[14] Motwani M (2019). Hierarchy of clinical manifestations in SAVI N153S and V154M mouse models. Proc Natl Acad Sci U S A.

[15] Ratiu JJ (2022). Loss of Zfp335 triggers cGAS/STING-dependent apoptosis of post-β selection thymocytes. Nat Commun.

[16] Gulen MF (2023). cGAS-STING drives ageing-related inflammation and neurodegeneration. Nature.

[17] Gaidt MM (2017). The DNA inflammasome in human myeloid cells is initiated by a STING-cell death program upstream of NLRP3. Cell.

[18] Khan IS (2014). Enhancement of an anti-tumor immune response by transient blockade of central T cell tolerance. J Exp Med.

[19] Xing Y (2016). Late stages of T cell maturation in the thymus involve NF-κB and tonic type I interferon signaling. Nat Immunol.

[20] Bennion BG (2020). STING gain-of-function disrupts lymph node organogenesis and innate lymphoid cell development in mice. Cell Rep.

[21] Gui X (2019). Autophagy induction via STING trafficking is a primordial function of the cGAS pathway. Nature.

[22] Liu D (2019). STING directly activates autophagy to tune the innate immune response. Cell Death Differ.

[23] Nedjic J (2008). Autophagy in thymic epithelium shapes the T-cell repertoire and is essential for tolerance. Nature.

[24] Mizushima N (2004). In vivo analysis of autophagy in response to nutrient starvation using transgenic mice expressing a fluorescent autophagosome marker. Mol Biol Cell.

[25] Klionsky DJ (2021). Guidelines for the use and interpretation of assays for monitoring autophagy (4th edition). Autophagy.

[26] Li L (2014). New autophagy reporter mice reveal dynamics of proximal tubular autophagy. J Am Soc Nephrol.

[27] Gump JM, Thorburn A (2014). Sorting cells for basal and induced autophagic flux by quantitative ratiometric flow cytometry. Autophagy.

[28] Ashby KM, Hogquist KA (2023). A guide to thymic selection of T cells. Nat Rev Immunol.

[29] Anderson MS (2005). The cellular mechanism of Aire control of T cell tolerance. Immunity.

[30] Stritesky GL (2013). Murine thymic selection quantified using a unique method to capture deleted T cells. Proc Natl Acad Sci U S A.

[31] Irving BA (1998). Thymocyte development in the absence of pre-T cell receptor extracellular immunoglobulin domains. Science.

[32] Muranski P (2008). Tumor-specific Th17-polarized cells eradicate large established melanoma. Blood.

[33] Bakhru P (2017). Combination central tolerance and peripheral checkpoint blockade unleashes antimelanoma immunity. JCI Insight.

[34] Ramanjulu JM (2018). Design of amidobenzimidazole STING receptor agonists with systemic activity. Nature.

[35] Breed ER (2019). Measuring thymic clonal deletion at the population level. J Immunol.

[36] Park J-E (2020). A cell atlas of human thymic development defines T cell repertoire formation. Science.

[37] Kadouri N (2020). Thymic epithelial cell heterogeneity: TEC by TEC. Nat Rev Immunol.

[38] Kooshesh KA (2023). Health consequences of thymus removal in adults. N Engl J Med.

[39] Savino W (2006). The thymus is a common target organ in infectious diseases. PLoS Pathog.

[40] Rosichini M (2023). SARS-CoV-2 infection of thymus induces loss of function that correlates with disease severity. J Allergy Clin Immunol.

[41] Albano F (2019). Insights into thymus development and viral thymic infections. Viruses.

[42] Bigley TM (2022). Disruption of thymic central tolerance by infection with murine roseolovirus induces autoimmune gastritis. J Exp Med.

[43] Lienenklaus S (2009). Novel reporter mouse reveals constitutive and inflammatory expression of IFN-beta in vivo. J Immunol.

[44] Bastard P (2021). Preexisting autoantibodies to type I IFNs underlie critical COVID-19 pneumonia in patients with APS-1. J Exp Med.

[45] Casanova J-L, Anderson MS (2023). Unlocking life-threatening COVID-19 through two types of inborn errors of type I IFNs. J Clin Invest.

[46] Martinez RJ (2023). Type III interferon drives thymic B cell activation and regulatory T cell generation. Proc Natl Acad Sci U S A.

[47] Ashby KM (2024). Sterile production of interferons in the thymus affects T cell repertoire selection. Sci Immunol.

[48] Münz C (2006). Autophagy and antigen presentation. Cell Microbiol.

[49] Long J (2020). Notch signaling protects CD4 T cells from STING-mediated apoptosis during acute systemic inflammation. Sci Adv.

[50] Gray DHD (2006). Developmental kinetics, turnover, and stimulatory capacity of thymic epithelial cells. Blood.

[51] Miyazaki M (2008). Thymocyte proliferation induced by pre-T cell receptor signaling is maintained through polycomb gene product Bmi-1-mediated Cdkn2a repression. Immunity.

[52] Larkin B (2017). Cutting edge: activation of STING in T cells induces type I IFN responses and cell death. J Immunol.

[53] Warner JD (2017). STING-associated vasculopathy develops independently of IRF3 in mice. J Exp Med.

[54] Garland KM (2022). Chemical and biomolecular strategies for STING pathway activation in cancer immunotherapy. Chem Rev.

[55] Hines JB (2023). The development of STING agonists and emerging results as a cancer immunotherapy. Curr Oncol Rep.

[56] Fenioux C (2023). Publisher correction: thymus alterations and susceptibility to immune checkpoint inhibitor myocarditis. Nat Med.

[57] Gajewski TF, Higgs EF (2020). Immunotherapy with a sting. Science.

[58] Wang L (2024). STING agonist diABZI enhances the cytotoxicity of T cell towards cancer cells. Cell Death Dis.

