# Supplementary material for: Activated STING in the thymic epithelium alters T cell development and selection leading to autoimmunity
Source: J Clin Invest. 2025 Jun 26;135(17):e180252. doi: 10.1172/JCI180252 (PMC12404769; doi:10.1172/JCI180252)
Supplement: Supplemental data [file jci-135-180252-s026.pdf]

Supplementary Figure 1

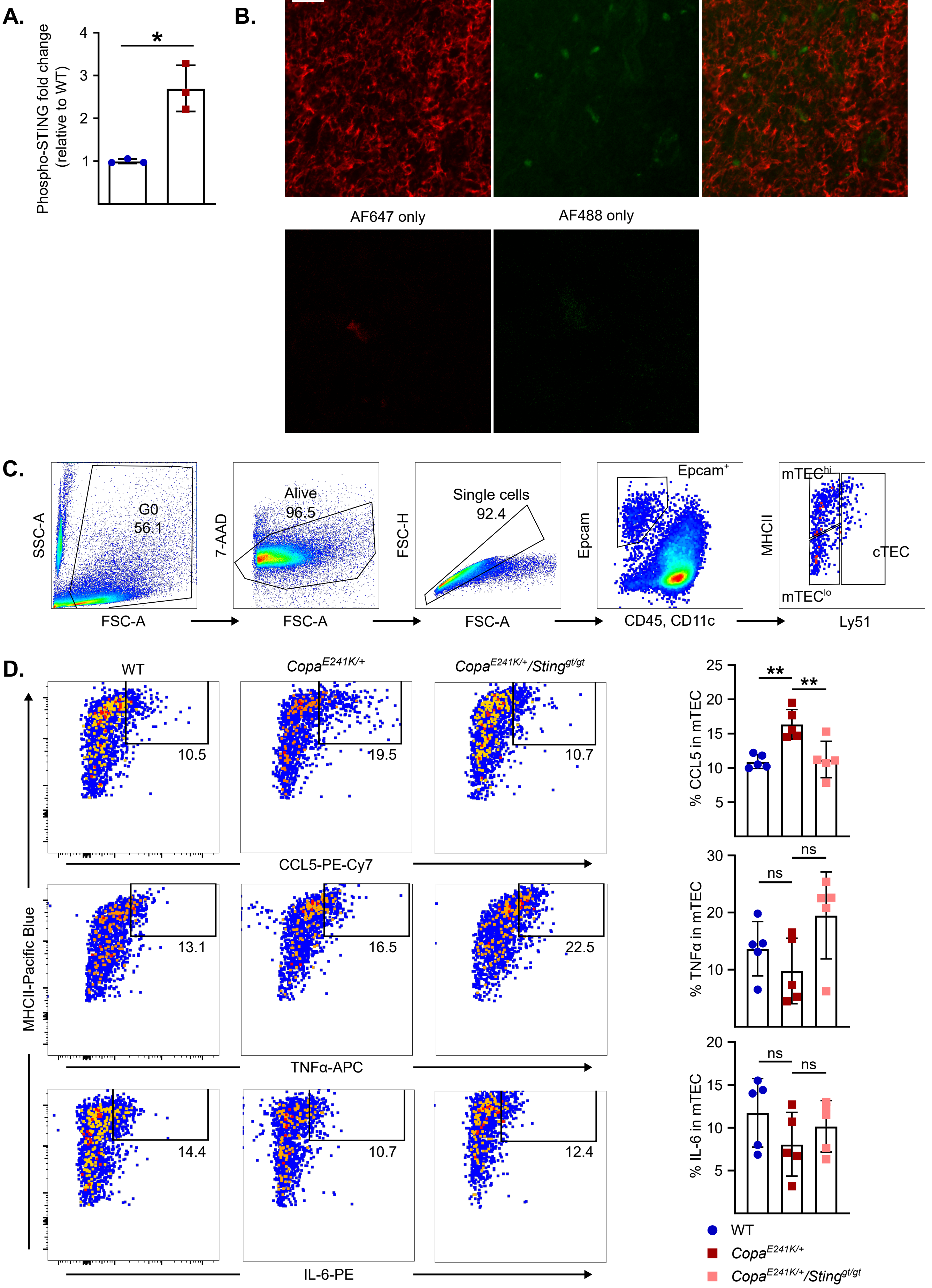

Supplementary Figure 1. (**A**) Quantitation of phosphorylated STING band density in thymic epithelial cells. Data is pooled from 3 independent immunoblots. (**B**) Representative immunofluorescent staining of keratin 5, pSTING, and secondary antibodies only on wild type thymic section. Image was captured with 40× objective. Scale bar represents 100 µm. (**C**) Gating strategy for flow cytometry analysis of thymic epithelial cells. (**D**) Flow cytometry analysis of CCL5, TNFα, and IL-6 in mTECs (WT, n = 5; *Copa*$^{E241K/+}$, n = 5; *Copa*$^{E241K/+}$/*Sting*$^{gt/gt}$, n = 5). Data are pooled from 2 independent experiments and presented as mean ± SD. Unpaired, parametric, two-tailed Student's t-test was used for statistical analysis in (A). One-way ANOVA with Bonferroni's multiple comparisons test was used in (D). $p < 0.05$ is considered statistically significant. ns: not significant.

Supplementary Figure 2

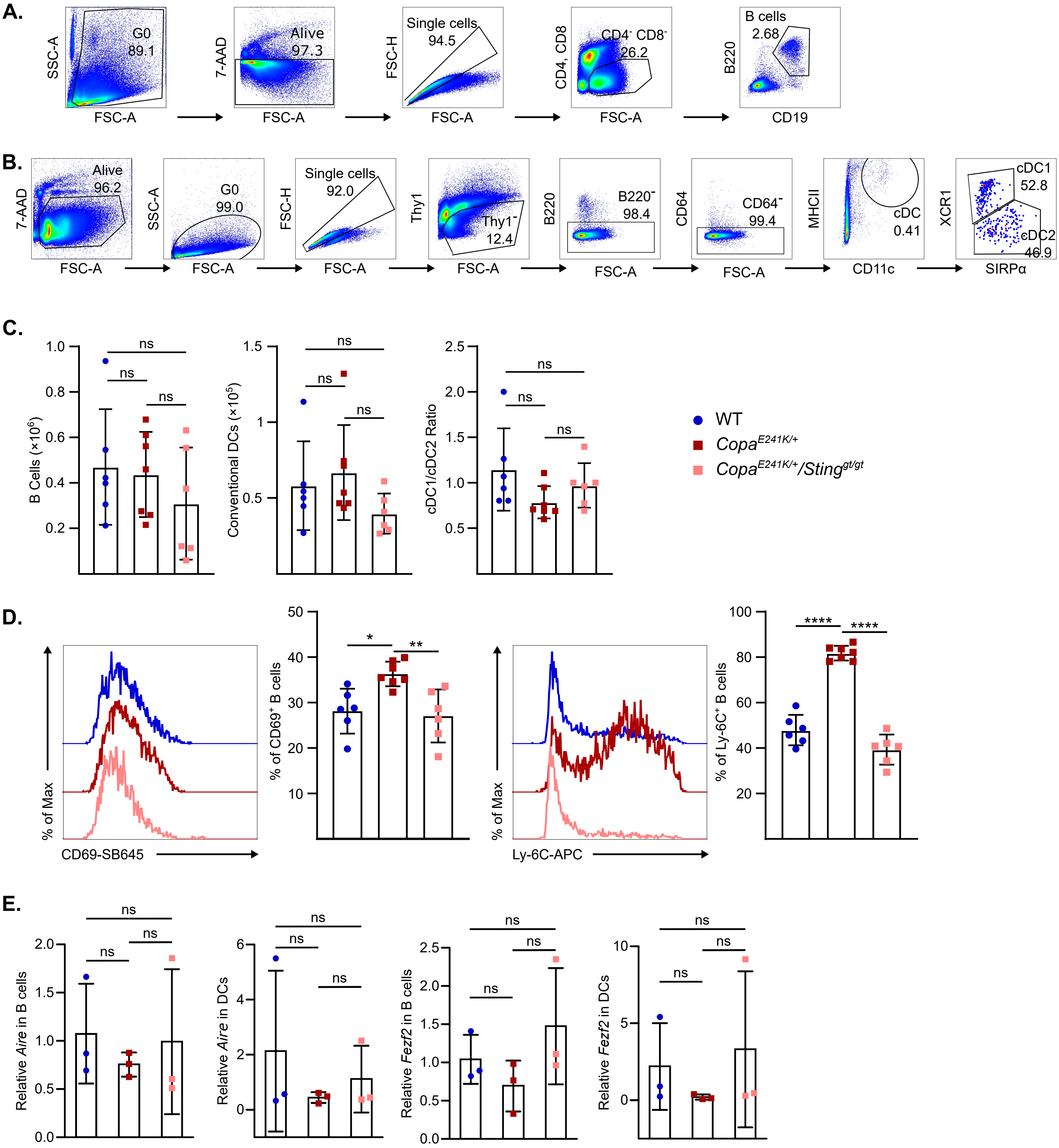

Supplementary Figure 2. Gating strategy for flow cytometry analysis of thymic (**A**) B cells and (**B**) conventional dendritic cells (cDCs). (**C**) Left: total count of B cells; middle: total count of cDCs; right: ratio of cDC1/cDC2 (WT, n = 6; *Copa*$^{E241K/+}$, n = 7; *Copa*$^{E241K/+}$/*Sting*$^{gt/gt}$, n = 6). Data are pooled from 2 independent experiments. (**D**) Left: representative histogram and quantitation from flow cytometry analysis of CD69^{+} B cells in the thymus; right: representative histogram and quantitation of thymic Ly-6C^{+} B cells (WT n = 6, *Copa*$^{E241K/+}$ n = 7, *Copa*$^{E241K/+}$/*Sting*$^{gt/gt}$ n = 6; data pooled from 2 independent experiments). (**E**) Quantitative PCR analysis of *Aire* and *Fezf2* transcripts in thymic B and dendritic cells (WT, n = 3; *Copa*$^{E241K/+}$, n = 3; *Copa*$^{E241K/+}$/*Sting*$^{gt/gt}$, n = 3). Data are presented as mean ± SD. One-way ANOVA with Bonferroni's multiple comparison test was used for statistical analysis. $p < 0.05$ is considered statistically significant. ns: not significant.

Supplementary Figure 3

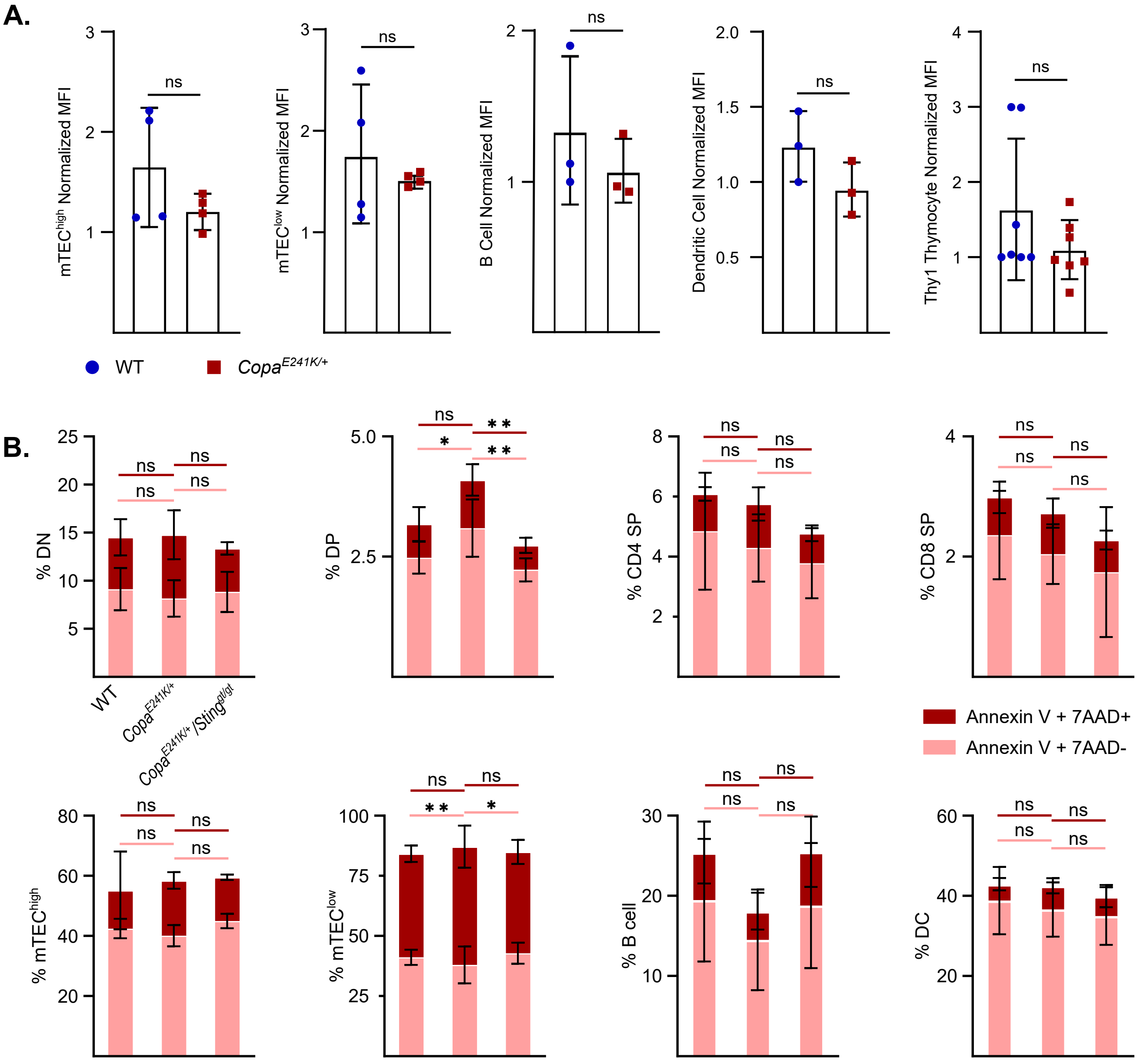

Supplementary Figure 3. (**A**) Normalized MFI of C12FDG staining of thymic populations $mTEC^{high}$ (n = 4), $mTEC^{low}$ (n = 4), B (n = 3), DC (n = 3), and Thy1 thymocytes (n = 7). (**B**) Quantitation of apoptosis via Annexin V and 7-aminoactinomycin D (7-AAD) staining in thymic populations (WT, n = 7; *Copa*$^{E241K/+}$, n = 8; *Copa*$^{E241K/+}$/*Sting*$^{gt/gt}$, n = 7) double negative, double positive, CD4 single positive, CD8 single positive, $mTEC^{high}$ (n = 4 for all), $mTEC^{low}$ (n = 4 for all), B (n = 6, 7, 6 respectively), and DC. Data are pooled from two independent experiments and presented as mean ± SD. Unpaired, parametric, two-tailed Student's t-test was used for statistical analysis in (A). One-way ANOVA with Bonferroni's multiple comparison test was used in (B). $p < 0.05$ is considered statistically significant. ns: not significant.

Supplementary Figure 4

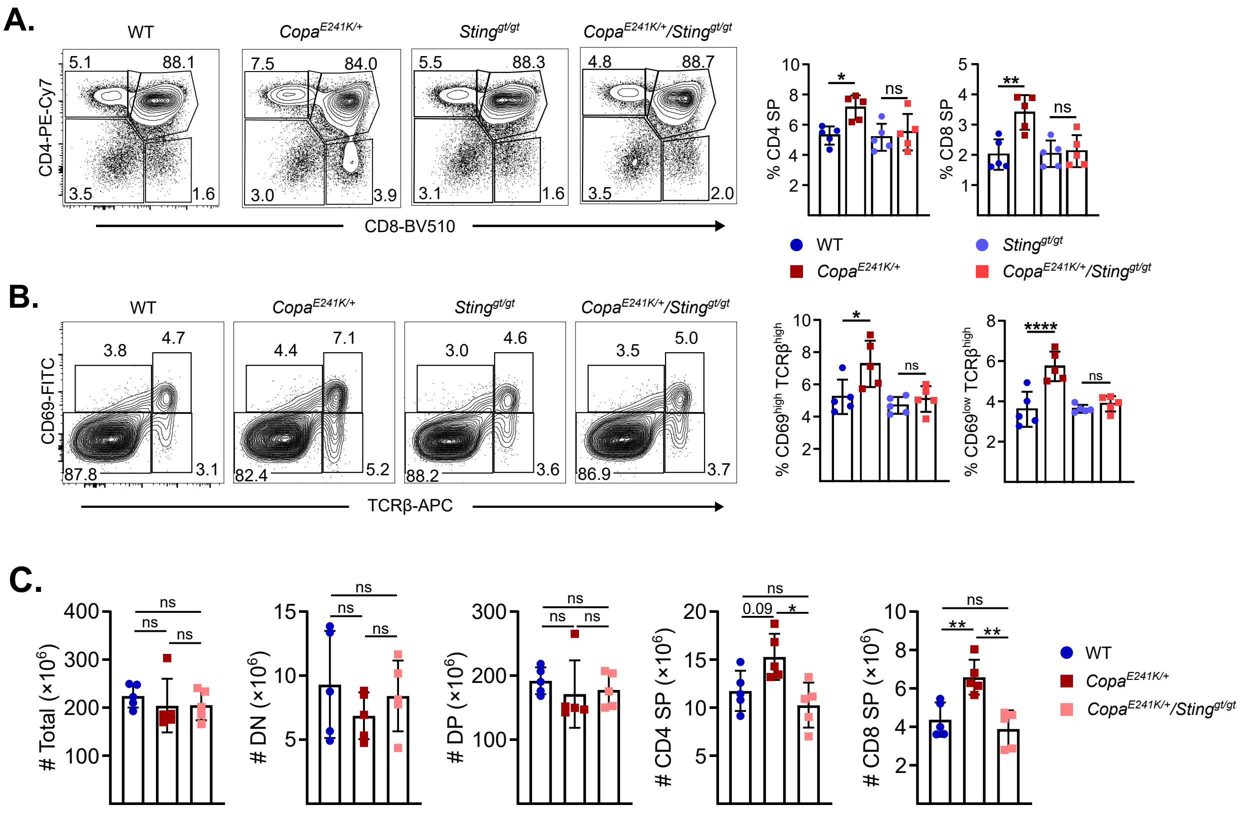

Supplementary Figure 4. (**A**) Left: CD4 and CD8 profile of thymocytes. Right: percentages of single positive CD4 and CD8 thymocytes in indicated mice. (**B**) Left: thymocyte expression of CD69 and TCR beta chain. Right: percentage of CD69^{high} TCRβ^{high} and CD69^{low} TCRβ^{high} thymocytes in indicated mice. (**C**) Thymocyte counts in indicated mice. Data are pooled from 2 independent experiments (n = 5 for all genotypes) and presented as mean ± SD. One way ANOVA with Bonferroni's multiple comparison test was used for statistical analysis. *p* < 0.05 is considered statistically significant. ns: not significant.

Supplementary Figure 5

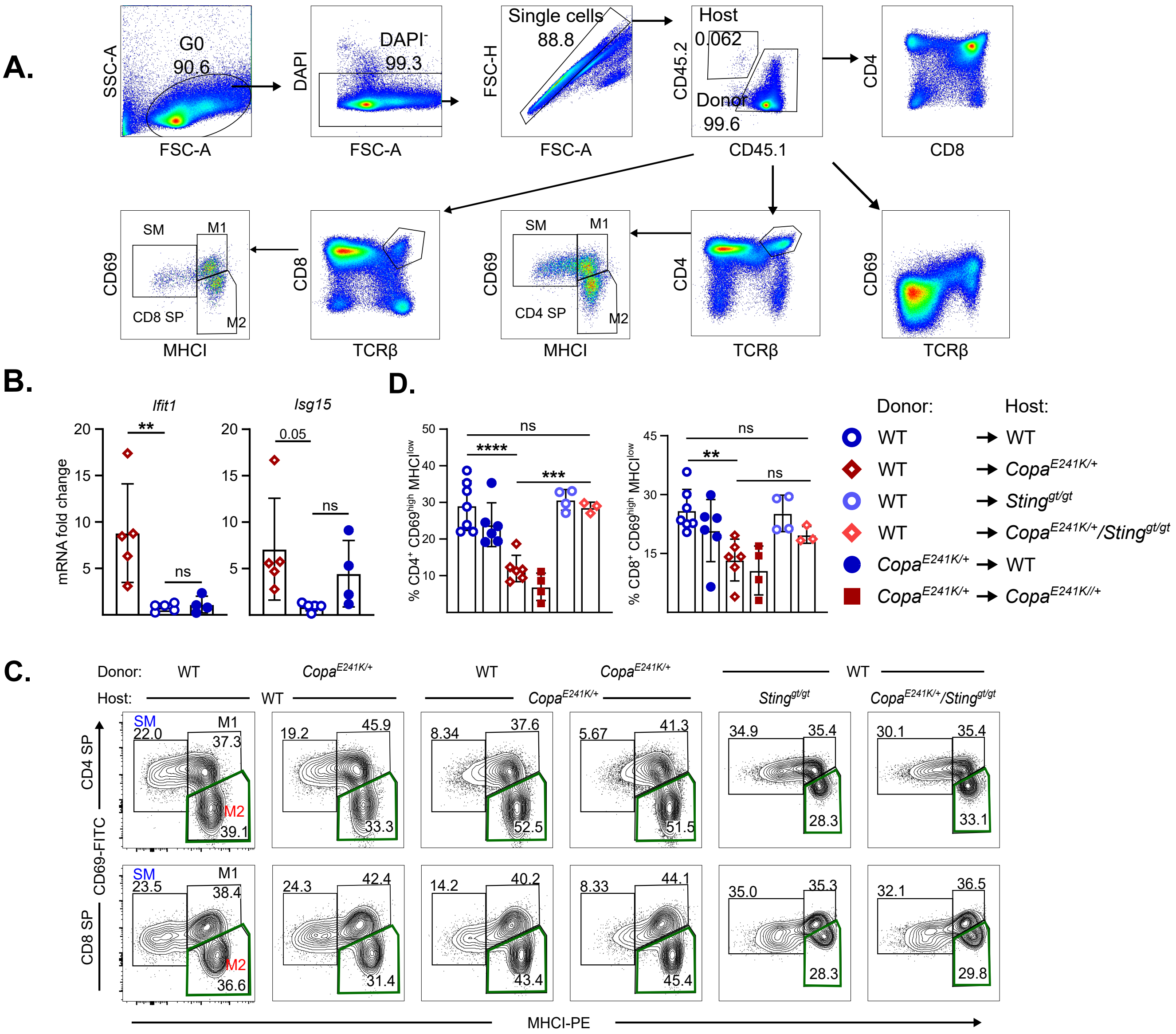

Supplementary Figure 5. (**A**) Gating strategy for CD4 and CD8 single positive thymocytes and subsequent CD69, TCRβ, and MHCI profiling in bone marrow chimeras. (**B**) Relative expression of interferon stimulated genes *Ifit1* and *Isg15* transcript in thymic stroma of indicated chimeras (WT→*Copa*$^{E241K/+}$, n = 5; WT→WT, n = 5; *Copa*$^{E241K/+}$→WT, n = 4). (**C**) Flow analysis of CD69 versus MHC-I on reconstituted single positive thymocytes in bone marrow chimeras indicating SM, M1 and M2 populations in CD4 and CD8 cells. (WT→WT, n = 7; WT→*Copa*$^{E241K/+}$, n = 6; *Copa*$^{E241K/+}$→WT, n = 6; *Copa*$^{E241K/+}$→*Copa*$^{E241K/+}$, n = 4; WT→*Sting*$^{gt/gt}$, n = 4; WT→ *Copa*$^{E241K/+}$/*Sting*$^{gt/gt}$, n = 3). (**D**) Percentages of CD69^{high} MHCI^{low} (semi-mature: SM) cells among the reconstituted CD4 and CD8 single positive thymocytes in BM chimeras. Data are pooled from 2 independent experiments and presented as mean ± SD. One way ANOVA with Bonferroni's multiple comparison test was used for statistical analysis. $p < 0.05$ is considered statistically significant. ns: not significant.

Supplementary Figure 6

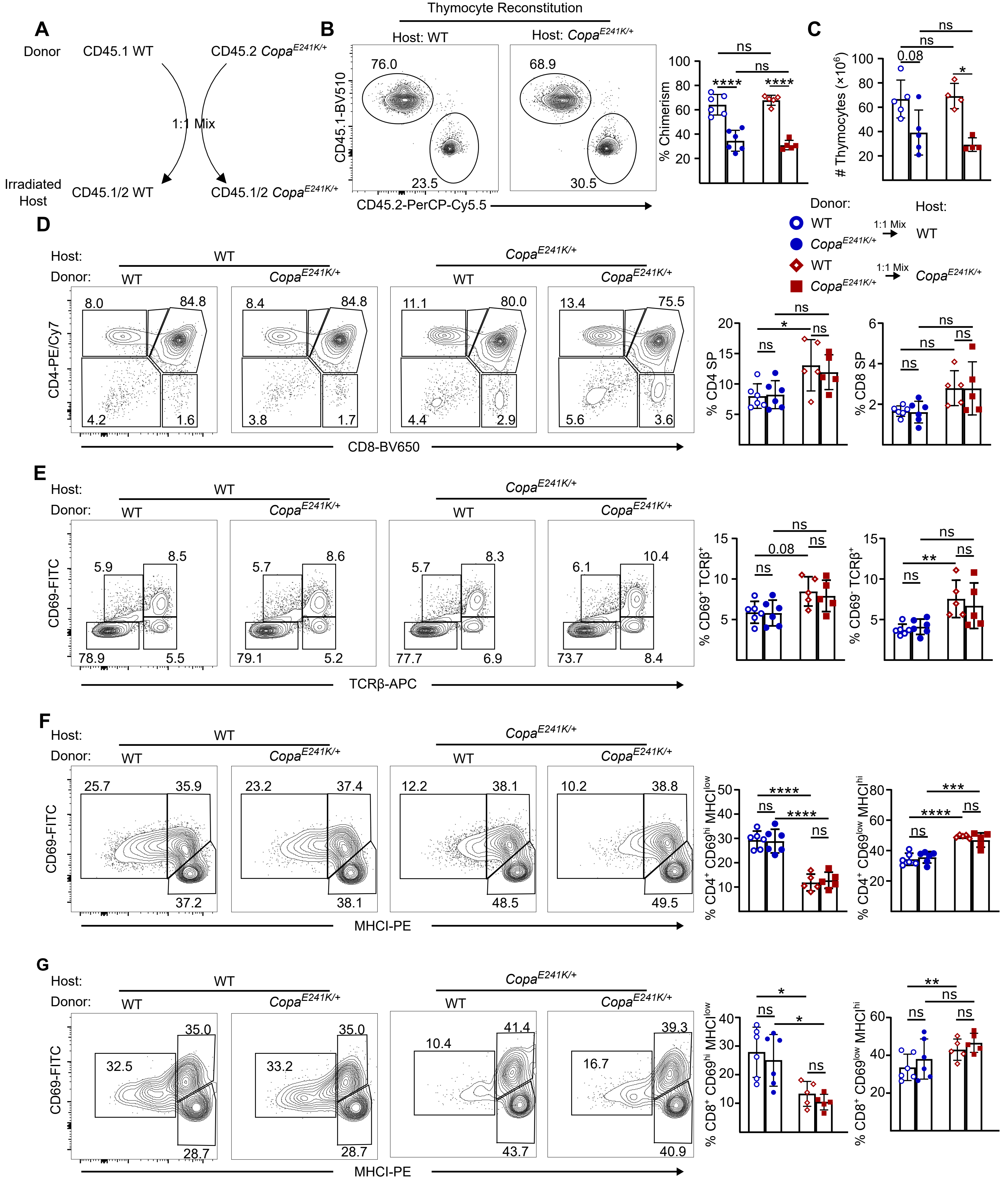

Supplementary Figure 6. (**A**) Schematic for generation of mixed chimeras using congenically marked donors and irradiated hosts (CD45.1 WT + CD45.2 *Copa*$^{E241K/+}$ → CD45.1/2 WT n = 6, CD45.1 WT + CD45.2 *Copa*$^{E241K/+}$ → CD45.1/2 *Copa*$^{E241K/+}$ n = 5). (**B**) Donor composition of reconstituted thymi in chimeras. Left: representative flow cytometry plot of CD45.1^{+} vs CD45.2^{+} thymocytes. Right: percentage of chimerism in hosts. (**C**) Total thymocyte count in reconstituted chimeras. (**D**) Left: CD4 and CD8 profile of reconstituted thymi. Right: Percentages of CD4 and CD8 single positive thymocytes in chimeras. (**E**) Characterizing selection of reconstituted thymocytes with CD69 and TCRβ expression. Analysis of thymocyte maturation via CD69 and MHCI in reconstituted (**F**) CD4 and (**G**) CD8 single positive cells. Data are pooled from 2 independent experiments and presented as mean ± SD. One-way ANOVA with Bonferroni's multiple comparison test was used for statistical analysis. $p < 0.05$ is considered statistically significant. ns: not significant.

Supplementary Figure 7

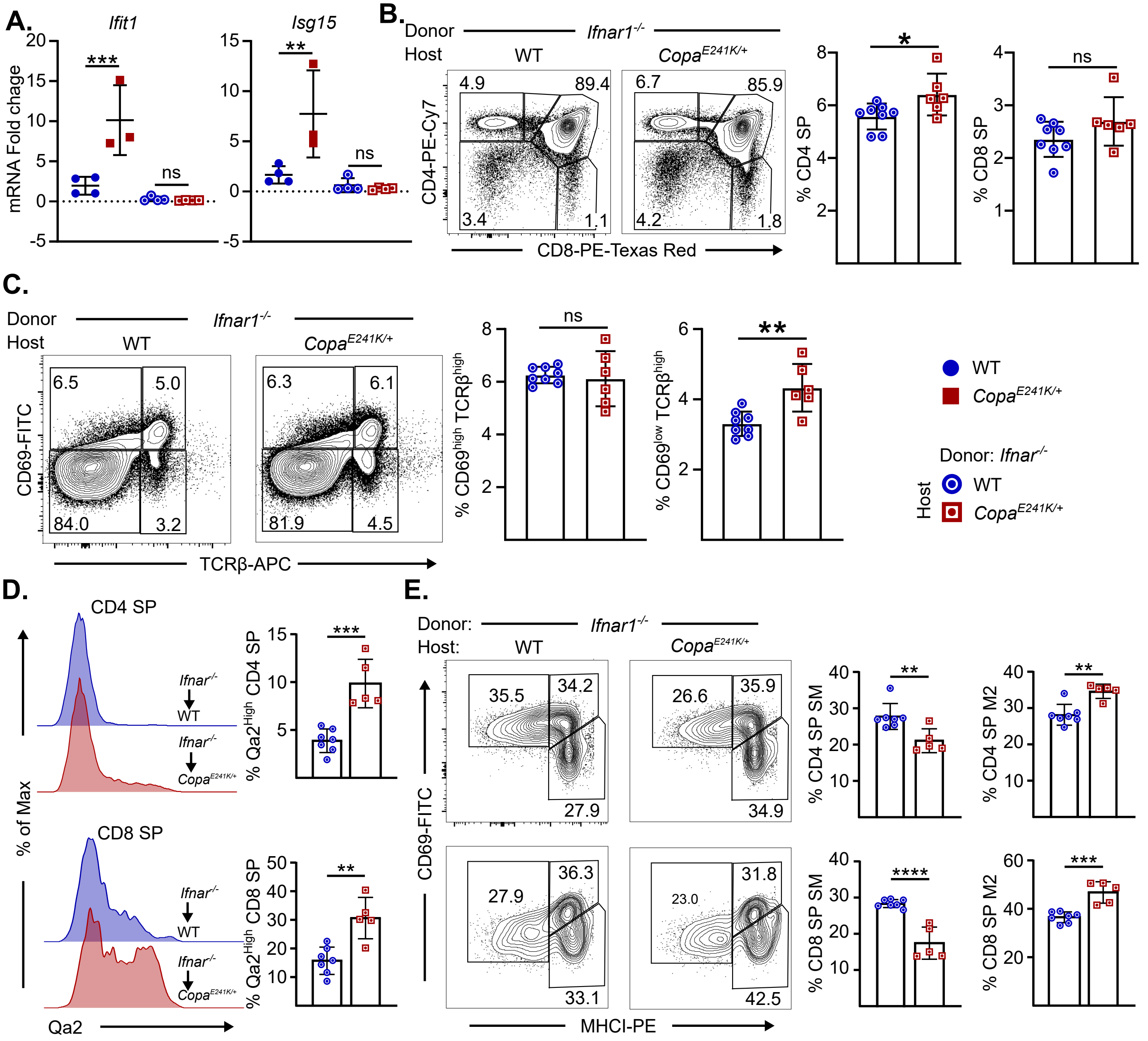

Supplementary Figure 7. (**A**) Relative transcript expression of interferon stimulated genes *Ifit1* and *Isg15* in thymic stroma of indicated mice (WT, n = 4; *Copa*$^{E241K/+}$, n = 4; *Ifnar1*$^{-/-}$→WT, n = 4; *Ifnar1*$^{-/-}$→*Copa*$^{E241K/+}$, n =4). (**B**) Left: CD4 and CD8 profile of thymocytes in *Ifnar1*$^{-/-}$ chimeras. Right: percentage of single positive CD4 and CD8 thymocytes in indicated chimeras (*Ifnar1*$^{-/-}$→WT, n = 7; *Ifnar1*$^{-/-}$→*Copa*$^{E241K/+}$, n = 5). (**C**) Left: CD69 and TCR beta chain profiling in reconstituted thymocytes of *Ifnar1*$^{-/-}$ chimeras. Right: percentages of CD69^{high} TCRβ^{high} and CD69^{low} TCRβ^{high} thymocytes in indicated chimeras (*Ifnar1*$^{-/-}$→WT, n = 7; *Ifnar1*$^{-/-}$→*Copa*$^{E241K/+}$, n = 5). Quantitation of (**D**) Qa2^{high} and (**E**) SM and M2 single positive thymocyte percentages in Ifnar-/- chimeras (*Ifnar1*$^{-/-}$→WT, n = 7; *Ifnar1*$^{-/-}$→*Copa*$^{E241K/+}$, n = 5). Data are from two independent experiments and presented as mean ± SD. One-way ANOVA with Bonferroni's multiple comparison test was used in (A) for statistical analysis. Unpaired, parametric, two-tailed Student's t-test was used in (B), (C), (D), and (E). *p* < 0.05 is considered statistically significant. ns: not significant.

Supplementary Figure 8

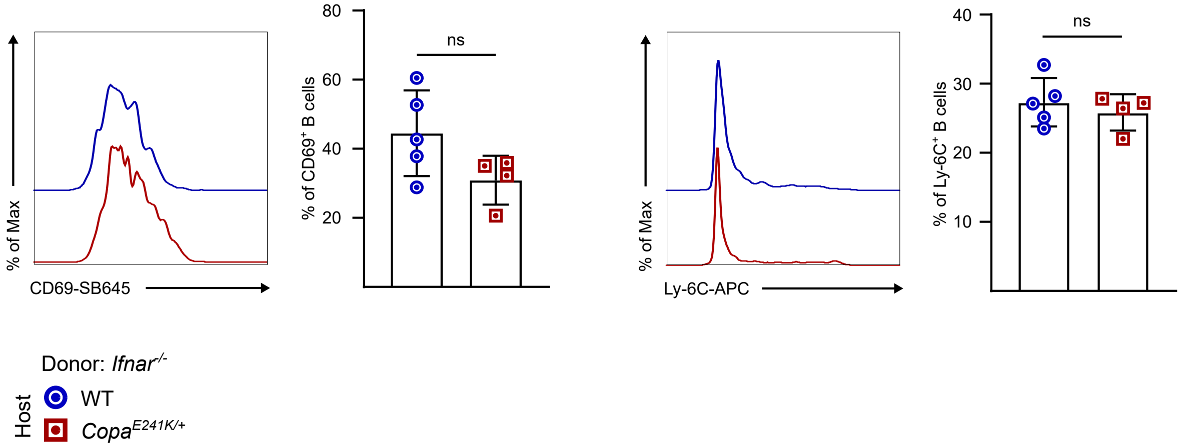

Supplementary Figure 8. Left: representative histogram and quantitation from flow cytometry analysis of CD69+ B cells in the thymus of Ifnar-/- bone marrow chimeras; right: representative histogram and quantitation of thymic Ly-6C+ B cells (*Ifnar*$^{-/-}$→WT, n = 5 *Ifnar*$^{-/-}$→*Copa*$^{E241K/+}$, n = 4; data pooled from 2 independent experiments). Data are presented as mean ± SD. Unpaired, parametric, two-tailed Student's t-test was used for statistical analysis. $p < 0.05$ is considered statistically significant. ns: not significant.

Supplementary Figure 9

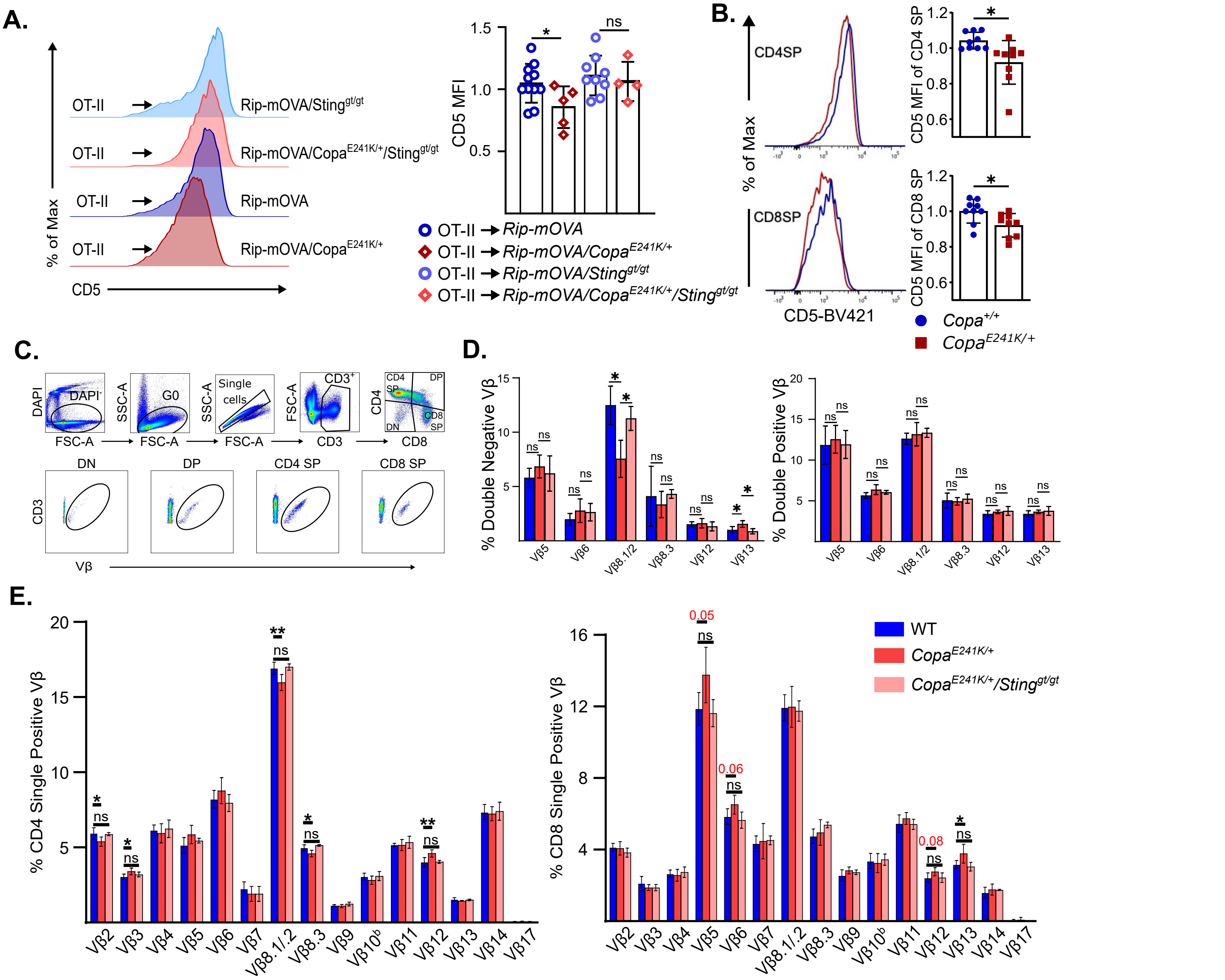

Supplementary Figure 9. (**A**) CD5 expression in Rip-mOVA/Copa/Sting chimeras that received OT-II bone marrow. Left: representative histogram of CD5 expression; and right: CD5 MFI for indicated chimeras (OT-II→WT, n = 11; OT-II→ *Copa*$^{E241K/+}$, n = 5; OT-II→*Sting*$^{gt/gt}$, n = 9; OT-II→*Copa*$^{E241K/+}$/ *Sting*$^{gt/gt}$, n = 4). Data are from 2 independent experiments. (**B**) CD5 expression in single positive thymocytes of WT and *Copa*$^{E241K/+}$ mice. Left: representative histogram of CD5 levels; and right: MFI for indicated mice (WT, n = 9; *Copa*$^{E241K/+}$, n = 9). Data are from 3 independent experiments. (**C**) Gating strategy for flow cytometry analysis of thymocytes and TCR Vβ expression. (**D**) Quantitation of TCR Vβ repertoire of CD4 and CD8 single positive thymocytes. (WT, n = 5; *Copa*$^{E241K/+}$, n = 4; *Copa*$^{E241K/+}$/*Sting*$^{gt/gt}$, n = 5). Data are from 2 independent experiments. (**E**) Quantitation of TCR Vβ repertoire of double positive and double negative thymocytes (WT, n = 2; *Copa*$^{E241K/+}$, n = 2; *Copa*$^{E241K/+}$/*Sting*$^{gt/gt}$, n = 2). Data presented as mean ± SD. Unpaired, parametric, two-tailed Student's t-test or one-way ANOVA with Bonferroni's multiple comparison test was used for statistical analysis. $p < 0.05$ is considered statistically significant. ns: not significant.

Supplementary Figure 10

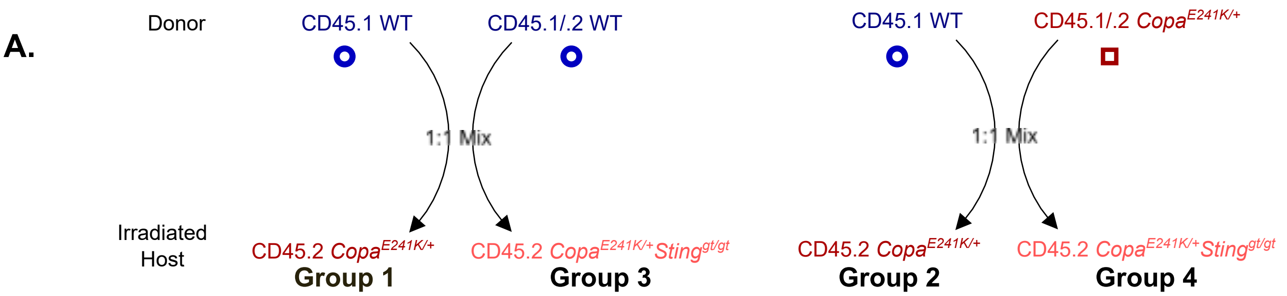

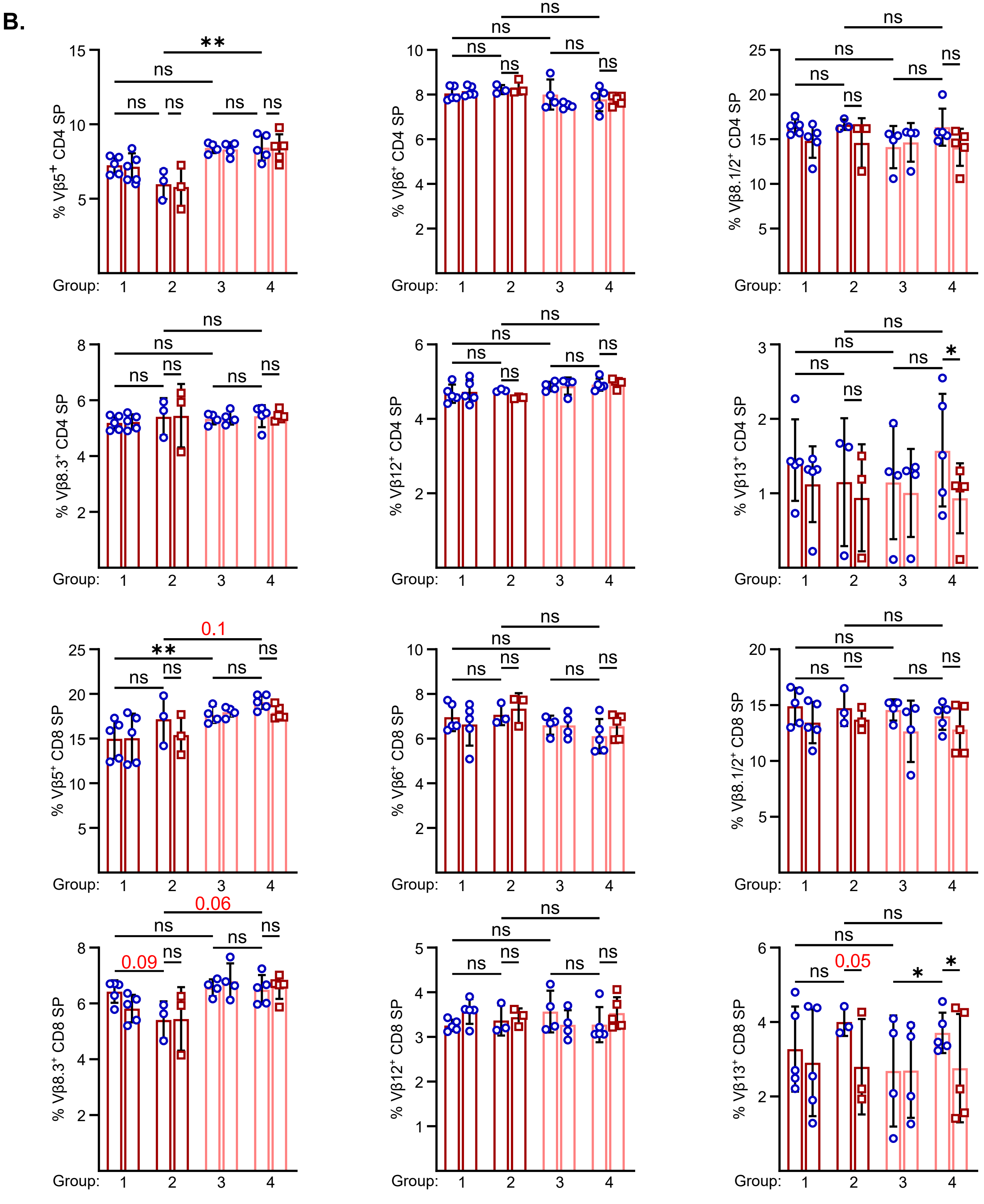

Supplementary Figure 10. (**A**) Schematic for generation of mixed chimeras using congenically marked donors and irradiated hosts (Group 1 n = 5, Group 2 n = 3, Group 3 n = 4, Group 4 n = 5). (**B**) Resulting TCR Vβ repertoire analysis for reconstituted CD4 and CD8 single positive thymocytes. Data presented as mean ± SD. Two-way ANOVA with Šidák's multiple comparisons test was used for statistical analysis. $p < 0.05$ is considered statistically significant. ns: not significant.

Supplementary Figure 11

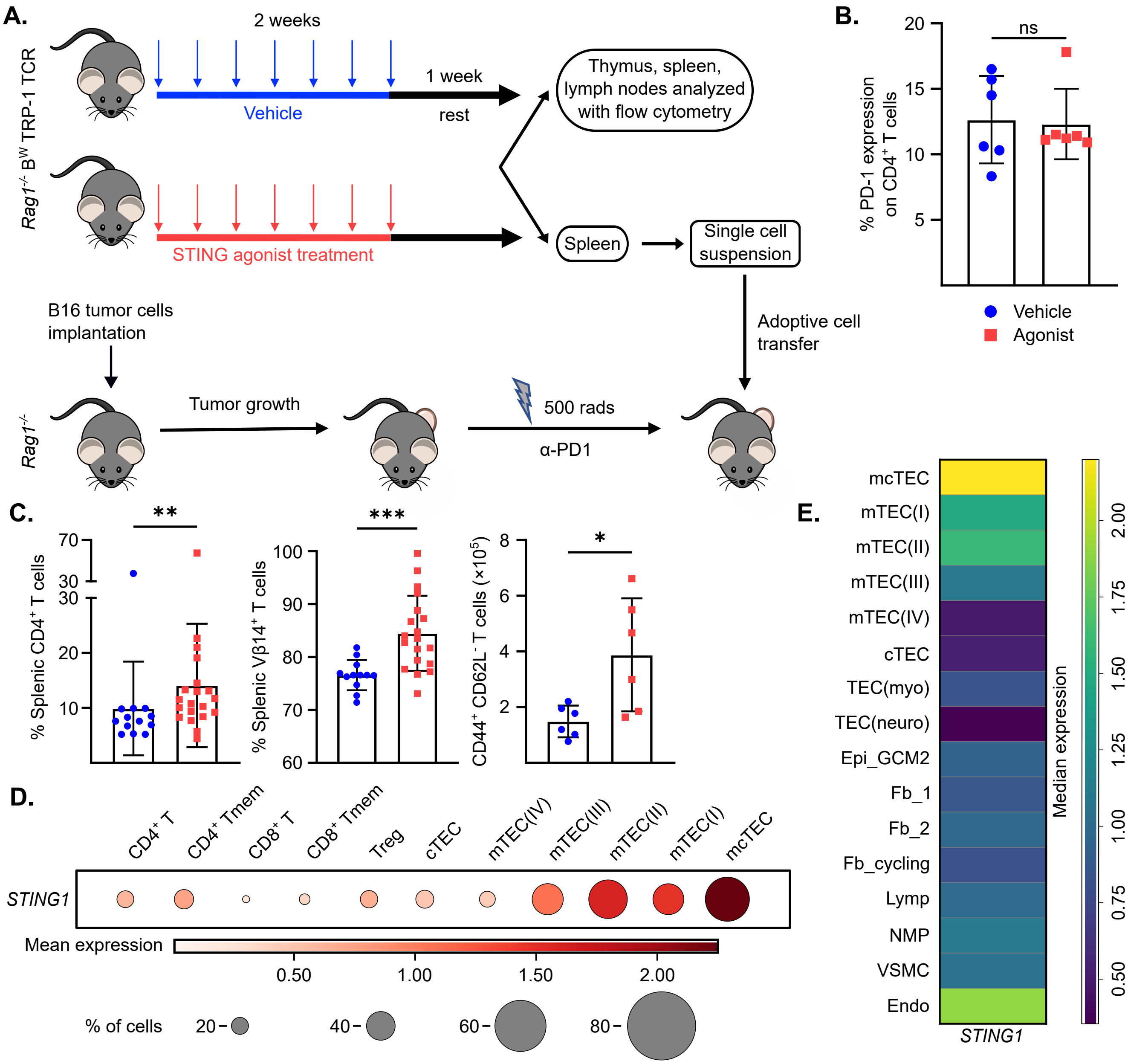

Supplementary Figure 11. (**A**) Schematic for administering vehicle or diABZI STING agonist to *Rag1*$^{-/-}$ *Tyrp1*$^{B-w/wt}$ TCR mice and B16 tumor cell inoculation with adoptive cell transfer into *Rag1*$^{-/-}$ mice. (**B**) Percentage PD-1 expression on peripheral CD4 single positive thymocytes (vehicle n = 6, agonist n = 6; data are pooled from 2 independent experiments). (**C**) Left: splenic percentage CD4 single positive thymocytes in treated *Rag1*$^{-/-}$ *Tyrp1*$^{B-w/wt}$ TCR mice (vehicle n = 13, agonist n = 20; data are pooled from 3 independent experiments). Middle: percentage of splenic Vβ14^{+} CD4 SP thymocytes (vehicle n = 12, agonist n = 19; data are pooled from 3 independent experiments). Right: absolute number of effector memory cells (vehicle n = 6, agonist n = 6; data are from 2 independent experiments). (**D**) Dot plot for *STING1* mean transcript expression and percent cells expressing transcript, comparing human thymocytes to thymic epithelial cells. (**E**) Matrix plot of median *STING1* transcript expression in human thymic stromal cells. Unpaired, parametric, two-tailed Student's t-test was used for statistical analysis. $p < 0.05$ is considered statistically significant. ns: not significant.

Supplementary Figure 12

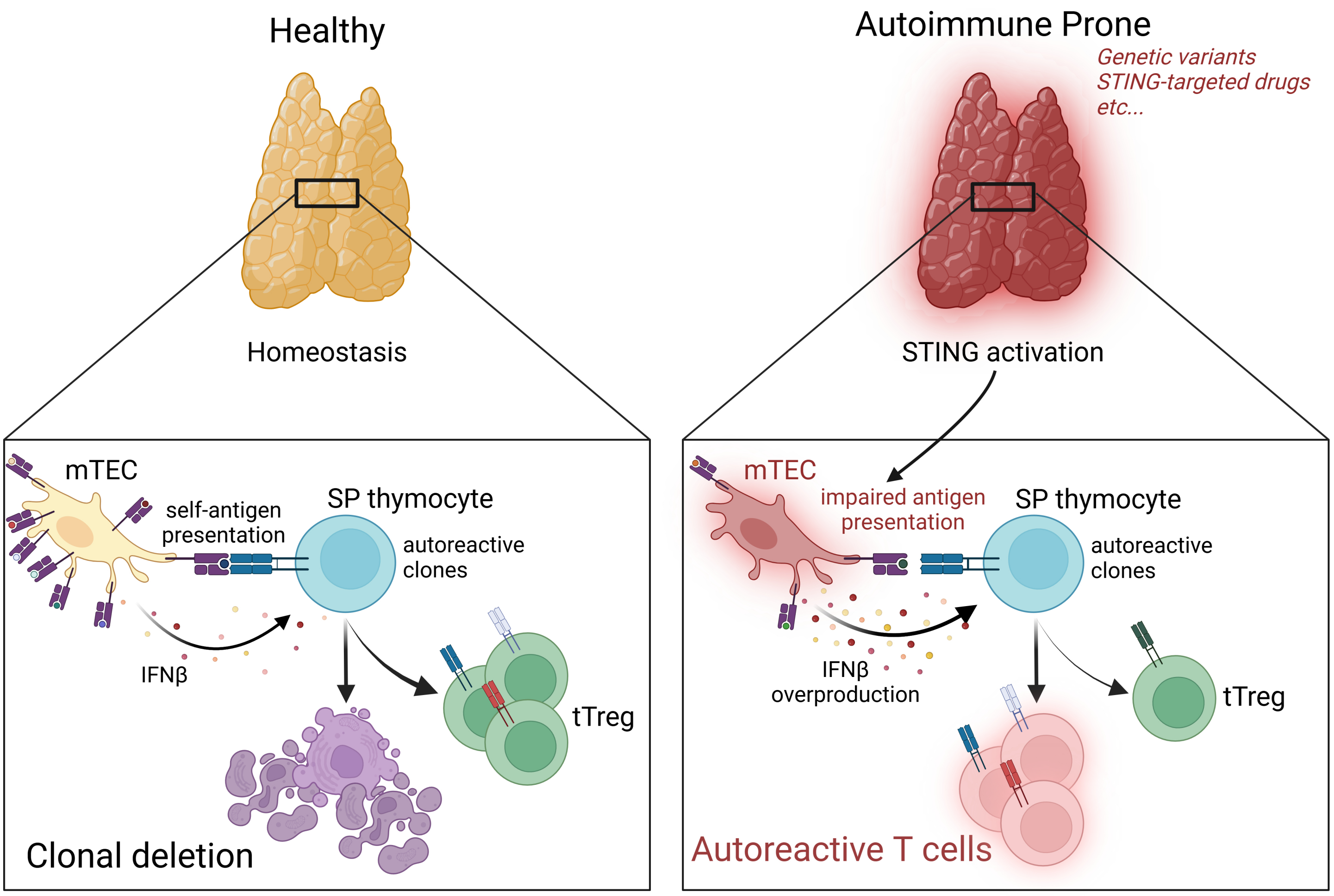

Supplementary Figure 12. A graphical summary showing that activation of thymic STING upregulates type I interferon signaling and impairs autophagic flux thereby alters thymocyte maturation and caused both a defect in negative selection and shift in the T cell repertoire.
