# Supplementary material for: Activated STING in the thymic epithelium alters T cell development and selection leading to autoimmunity
Source: J Clin Invest. 2025 Jun 26;135(17):e180252. doi: 10.1172/JCI180252 (PMC12404769; doi:10.1172/JCI180252)

Full unedited blots for Figure 1A

Phosphorylated STING

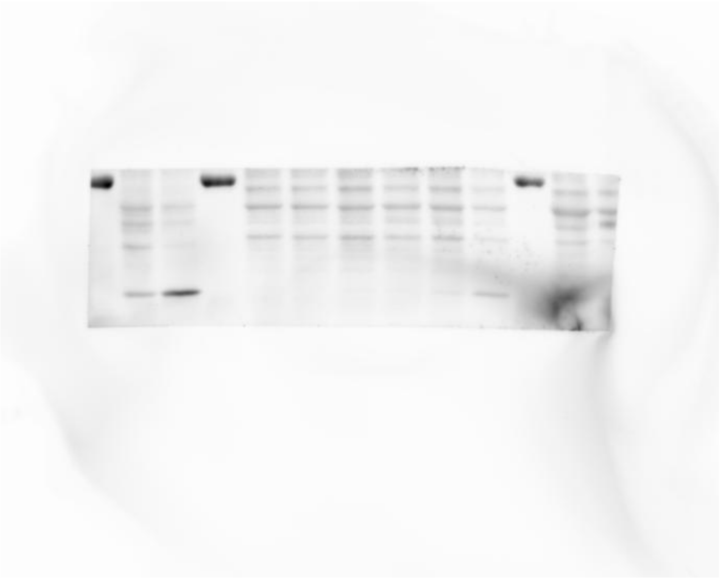

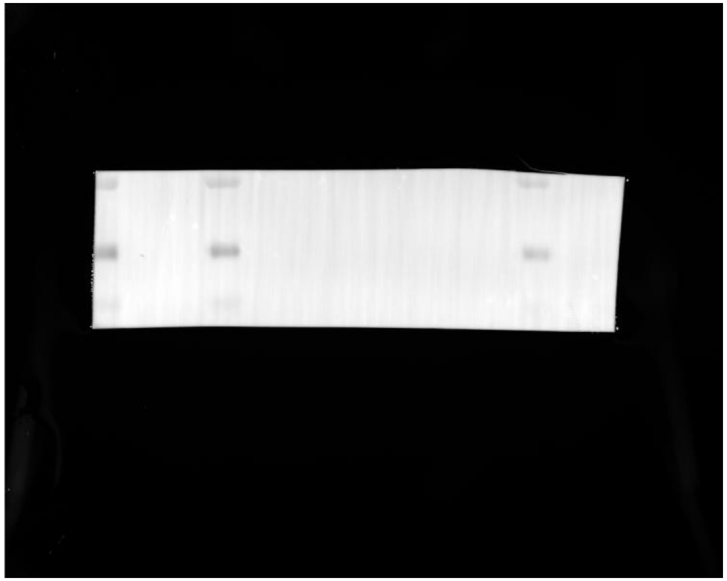

GAPDH

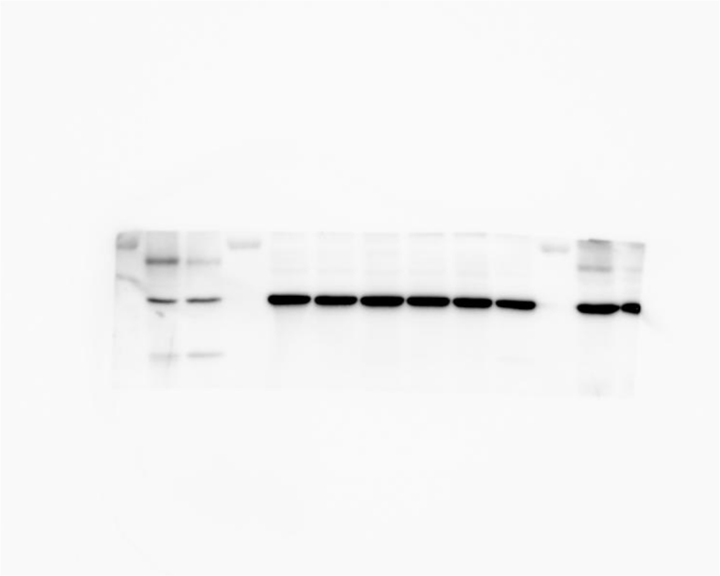

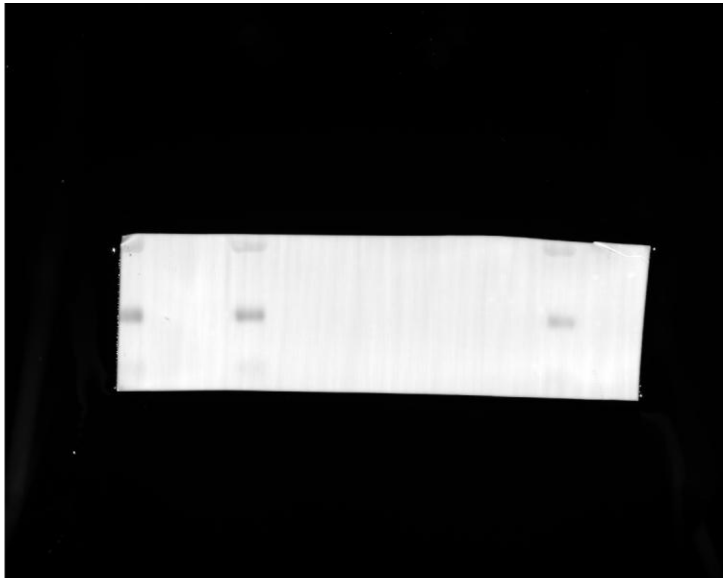

Full unedited blots for Figure 3B

LC3II

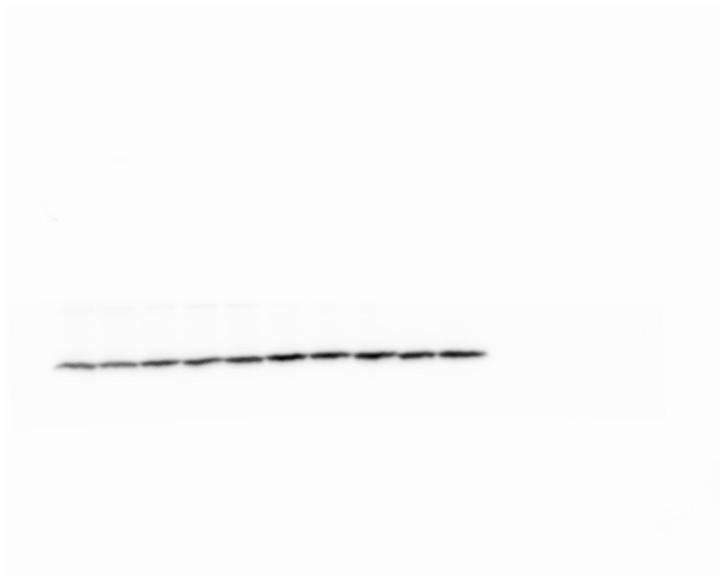

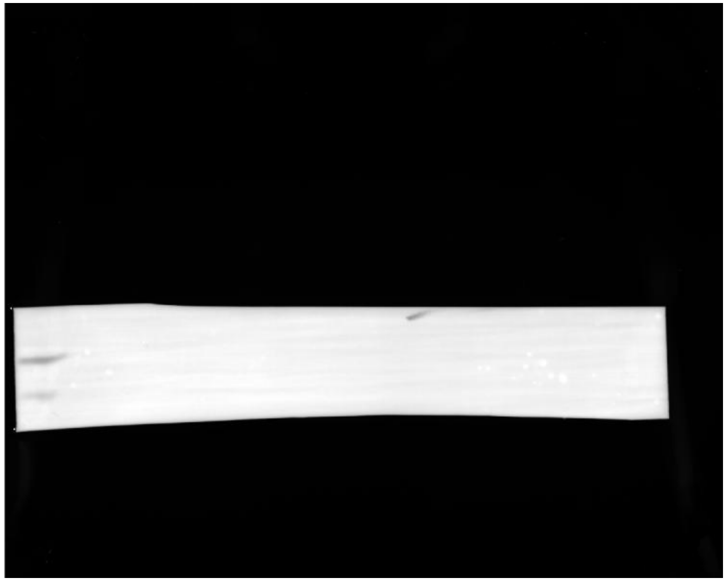

GAPDH

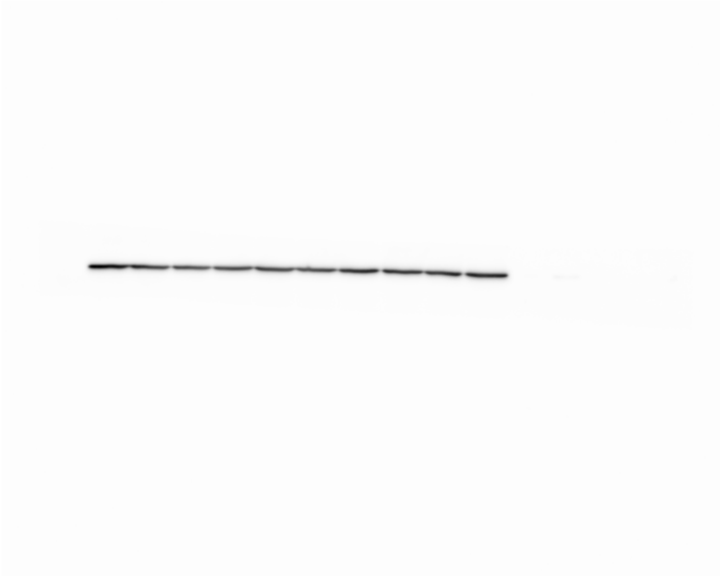

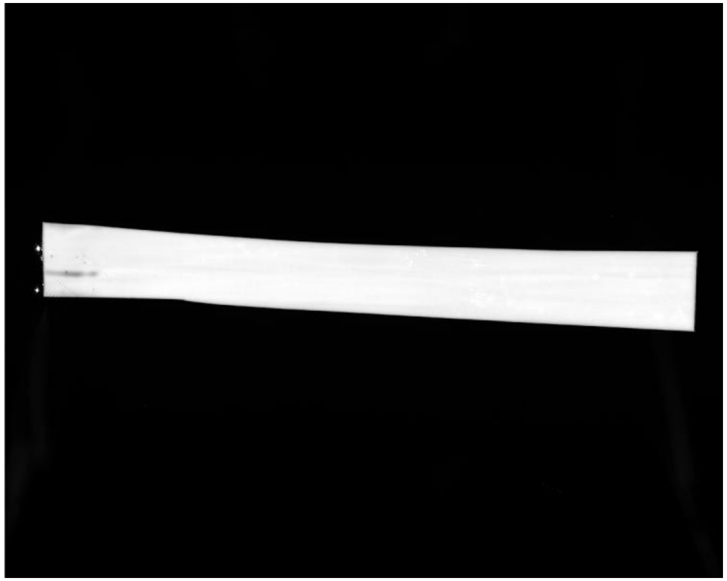

Supplement: Unedited blot and gel images [file jci-135-180252-s027.pdf]
